# Material and carbon implications of future mobility infrastructure expansion around the world

**DOI:** 10.1007/s44498-026-00132-x

**Published:** 2026-07-18

**Authors:** André Baumgart, Gamze Ünlü, Benedikt Grammer, Aneeque Javaid, Florian Maczek, Fridolin Krausmann, Volker Krey, Dominik Wiedenhofer

**Affiliations:** 1 BOKU University Vienna, Schottenfeldgasse 29, 1070 Vienna, Austria; 2 International Institute for Applied Systems Analysis, Schloßplatz 1, 2361 Laxenburg, Austria

**Keywords:** Mobility infrastructure, Material stocks, Roads, Embodied emissions, Built environment, Transport

## Abstract

Global mobility infrastructure is expected to expand significantly, especially in the Global South, potentially requiring a substantial share of the remaining carbon budget. However, the resource use and carbon emissions associated with the anticipated expansion of roads, railways, and other mobility infrastructure, as well as the potential of mitigation measures, have so far not been explored systematically. Using high-resolution infrastructure and mobility data, we assess seven demand- and four supply-side circular economy and decarbonization measures aiming to mitigate resource use and associated embodied CO_2_ emissions. We find that without demand reduction, infrastructure stocks could double or triple by 2060, with cumulative emissions reaching 10 to 20 GtCO_2_. We find that a global shift to rail would increase embodied emissions to 37 GtCO_2_ due to carbon-intensive materials. Combining mobility reduction with enhanced recycling and industrial decarbonization can cut emissions by 63% while providing adequate global infrastructure. This would help to offset increases in emissions from public transport infrastructure expansion required to reduce operational emissions.

**Supplementary Information:**

The online version contains supplementary material available at 10.1007/s44498-026-00132-x.

## Introduction

Mobility is vital for the participation in society by enabling the transportation of people and goods from one place to another, utilizing mobility infrastructure networks to connect places. So far, future changes in modal choice, technology transitions (Hao et al., 2016), electrification of vehicles (Speizer et al., 2024), as well as energy and material efficiency of vehicles (Cox et al., 2020; Sacchi et al., 2022) have received substantial attention, especially in the light of growing mobility demand around the world (Jaramillo et al., 2022; Miskolczi et al., 2021; Yan et al., 2014). Passenger and freight transportation is estimated to double by 2050, with the majority of additional kilometers occurring in Northeast- and Southern Asia (ITF, 2023; Khalili et al., 2019). However, the pivotal role of mobility infrastructure networks enabling mobility, and in particular their future expansion pathways and the associated resource use and carbon emissions, have mostly been overlooked in literature.

Mobility infrastructure encompasses all road and rail networks, including tunnels and bridges, as well as associated infrastructure (parking, fueling), and airport runways. While early work aimed to quantify road and railway networks around the world based on official statistics (Canning & Farahani, 2007), later research showed massive under-estimations especially of road networks globally (Barrington-Leigh & Millard-Ball, 2017). Recent research, therefore leveraged crowd-sourced data from OpenStreetMap – for which road network coverage was shown to be more than 80% complete (Barrington-Leigh & Millard-Ball, 2017) – to quantify currently existing mobility networks and their material requirements. Those studies yield estimates of 180 to 440 Gt of materials contained in 24 to 82 million km of mobility infrastructure networks, the majority being paved roads (Rousseau et al., 2022; van Engelenburg et al., 2024; Wiedenhofer et al., 2024).

Previous work also shows that existing mobility infrastructure is highly unequally distributed around the world. Densely populated, lower-income world regions have material stocks below 5 metric tons per capita, or less than 1 m/cap in length, while low-density high-income regions reach almost 500 t/cap in stocks and more than 230 m/cap in length (Wiedenhofer et al., 2024). This implies unequal access to mobility services and raises the question of what constitutes a sufficient level of mobility for all and how much material is required to enable such a level (Rao & Min, 2018; Vélez-Henao & Pauliuk, 2023). The Sustainable Development Goals utilize the rural access index to measure the availability of all-season mobility infrastructure. A quasi-universal access to roads across the globe is estimated to require an additional 4 million km of road (Wenz et al., 2020). Further work shows that sufficient and decent mobility levels can be achieved with mobility infrastructure stocks of around 100 t/cap – a threshold where wellbeing gains begin to saturate with material stock intensity. It is surpassed by roughly 20% of countries (Virág et al., 2022a, 2022b). However, much of the infrastructure expansion is driven by increasing mobility demand, and vice versa – a dynamic known as induced demand. This is evident, for example, in large infrastructure projects opening new trade routes. A prominent example is the ‘Belt and Road’ initiative, connecting economic centers across Eurasia (Thomas et al., 2024) and thus enabling growing freight transport. With increasing mobility demand, further expansion of infrastructure seems inevitable, particularly in connection with a ‘catch-up’ of economies of the Global South. While roads and railway lines have long service lives before they require replacement, they are continuously maintained (Grossegger et al., 2024; Saxe & Kasraian, 2020). This makes mobility infrastructure slow to change and, considering that the expansion of settlements and mobility infrastructure commonly extends already existing infrastructure networks, brings substantial path dependencies. Therefore, a fundamental understanding of the scale, structure and environmental implications of anticipated mobility infrastructure expansion is necessary, especially regarding the potentials of mitigation measures aiming to reduce material demand and carbon emissions, while providing sufficient mobility infrastructure for all.

From a systems-oriented, socio-metabolic research perspective, both vehicles and mobility infrastructure such as roads or railway lines are regarded as material stocks (Krausmann et al., 2017; Weisz et al., 2015) the latter accounting for one third of global technosphere mass (Galbraith et al., 2025). Complementing direct operational emissions of vehicles with the embodied emissions of infrastructure stocks therefore enables a holistic understanding of the overall environmental burdens of the mobility sector. Recent studies mapped currently existing mobility infrastructure stocks (Rousseau et al., 2022; van Engelenburg et al., 2024; Wiedenhofer et al., 2024). However, while scenarios for vehicle fleets have been developed (Hao et al., 2016; Pauliuk et al., 2021), insights into future mobility infrastructure expansion are missing. As a result, existing mobility and climate scenarios lack a consistent representation of how projected mobility demand translates into physical infrastructure expansion and its associated material and embodied carbon requirements.

To address these shortcomings, we herein present exploratory ‘what-if’ scenarios on global material stocks and flows and embodied carbon emissions due to global mobility infrastructure expansion and maintenance. These scenarios use simplified relationships to project the potential scale of future infrastructure, focusing on the assessment of the impact of certain circularity measures rather than on exact baseline stock projections. Our analysis covers the years 2024 to 2060 as well as all road- and rail-based infrastructure, airport runways, as well as parking and fueling infrastructure. We exclude the rolling stock of motor vehicles and trains in this assessment, which have been investigated elsewhere (Pauliuk et al., 2021).

## Methods

We introduce the biophysical dynamic stock-flow model of Global Mobility Infrastructure Stocks (GLOMIS), which is linked with the integrated assessment model MESSAGEix-Materials to dynamically quantify embodied emissions, i.e., emissions associated with material production. By combining energy system modelling with economy-wide material flow analysis, MESSAGEix-Materials models the life cycle of materials from extraction, through industrial processing stages, to waste treatment and recycling (Ünlü et al., 2024).

**Fig. 1:**
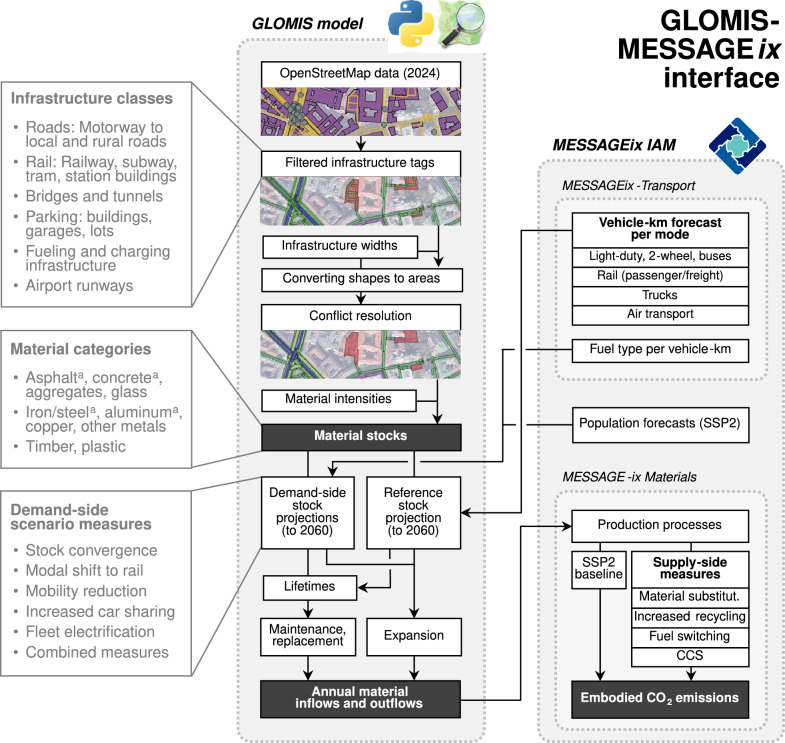
Model framework showing GLOMIS and MESSAGEix model processes and interlinkages. Model scope encompassing infrastructure classes, material categories, and demand-side scenario measures shown in the left column. *LDV* Light-duty vehicle, *CCS* carbon capture and storage, *SSP2* Shared socioeconomic pathways ‘Middle-of-the-road’ scenario. ^a^Materials for which embodied CO_2_ emissions are quantified in MESSAGEix-Materials

Figure 1 illustrates the five-step workflow used to model future mobility infrastructure material stocks, flows, and associated carbon emissions. First, global mobility infrastructure was mapped at 10 × 10 m resolution using OpenStreetMap (OSM) data and harmonized into consistent infrastructure classes. Second, vectorized geometries were integrated with class-specific material intensities and widths to estimate material stocks for the base year 2024. Third, using mobility demand projections as a proxy, future material stocks until 2060 were estimated. Fourth, annual material inflows and outflows were derived from annual net changes in stocks and infrastructure-specific average lifetimes. Finally, derived material flows were used as input to the MESSAGEix-Materials model to calculate embodied emissions from material production. Additional methodological details, data sources, scenario assumptions, uncertainties, and model limitations are provided in Supplementary Information SI1.

### Infrastructure areas

Mobility infrastructure areas per class were derived from open-access OSM data for 2024, obtained from Geofabrik (2024) and processed following established methods (van Engelenburg et al., 2024; Wiedenhofer et al., 2024). Relevant features were filtered and aggregated into 37 infrastructure classes, including various road types and rail-based systems, bridges, tunnels, and parking, fueling and charging infrastructure. Data was manually checked for inconsistencies, with class overlaps resolved using hierarchy-based rules. Non-polygonal OSM features (lines, points) were converted to areas using region-specific average widths collected from literature. While road widths might vary across regions, a manual validation using satellite imagery showed that assumptions were adequate for all road classes. Airport runways were derived from a specialized dataset with more complete surface material information (OurAirports, 2024).

### Material stocks

The material stock *MS* for the baseline year was calculated using Eq. 1, with *A* referring to the area per infrastructure class *i* in year *t*, *W* referring to the infrastructure width, and *MI* to the material intensity (MI) per material *m*.

1 $${MS}_{m,t}={\sum_{i}({A}_{i,t}{\times MI}_{i,m})}$$

MI factors were sourced from a previous study (Wiedenhofer et al., 2024) and expanded herein to accommodate additional infrastructure classes. Ten materials are covered: iron/steel, aluminum, copper, other metals (a mix class of various metals occurring in small amounts, often not classified consistently in literature), aggregates (sand and gravel, excluding aggregates embodied in asphalt and concrete), concrete, glass, asphalt, plastic, and timber.

In a reference scenario, material stocks were projected to the year 2060 using MESSAGEix-Transport (IIASA, 2024a) vehicle-km projections for 12 geographical MESSAGEix regions and seven transport modes as proxy (see Figure S5 in Supplementary Information SI1). This was done by matching material stocks with transport mode-specific vehicle-km data based on the assumed main vehicle usage of the respective infrastructure type (see Table S5 in Supplementary Information SI1). Consequently, for each country and infrastructure type, the future trajectory of a corresponding MESSAGEix region and combination of transport mode vehicle-km was used to project infrastructure stocks to the year 2060. Infrastructure stocks were assumed to not decrease over time, meaning that though vehicle-km might decrease in certain years, the model prevents stocks from doing so. Mobility infrastructure stocks have long lifetimes and are rarely decommissioned prematurely, instead becoming hibernating stocks (Grossegger et al., 2024; Hashimoto et al., 2009). This assumption may moderately overestimate long-term stocks but avoids implausible rapid downsizing of infrastructure.

This approach represents a ‘what-if’ scenario rather than a deterministic forecast, as the relationship between mobility demand and infrastructure expansion is complex and often non-linear. Infrastructure may be expanded in anticipation of demand or used more intensively until capacity limits are reached. Regional differences in road stock per vehicle-km (Figure S6a in Supplementary Information SI1) reflect varying utilization intensities and temporal lags. The model therefore applies a simplified linear relationship as a transparent proxy to explore potential future material stocks under different assumptions. To further test the sensitivity of our results to this linearity assumption, we apply caps to above-average intensity regions at mean, 25th, and 5th percentile levels (Figure S6b in Supplementary Information SI1), representing futures where infrastructure is utilized more intensively before expansion. Under these capped scenarios, global material stocks in 2060 range from 10 to 35% below reference levels, indicating that our reference estimates are likely conservative upper bounds.

### Prospective scenarios

To model possible trajectories of future mobility infrastructure stock development, we quantify future material demand and carbon emissions as well as mitigation potentials of six additional demand- and four supply-side measures (see Table 1).

**Table 1:** Demand- and supply-side scenarios, underlying circularity measures and respective sources for assumptions

Scenario	Description	Infrastructure-relevant assumptions	Sources
*Reference*	Reference scenario (no strategies)	Current stocks coupled with SSP2-regional vehicle-km forecasts to project to 2060	MESSAGEix-Transport projection (IIASA, 2024a)
*StockConv*	Convergence of total infrastructure area to decent infrastructure level	Convergence to ~ 1,110 m^2^/cap total infrastructure area by 2060. This threshold corresponds to the level where Human Development Index (HDI) – used a proxy for well-being – is 80% (threshold for high HDI) and HDI begins to saturate (see Figure S7 in Supplementary Information SI1). Shares of individual infrastructure classes in 2060 based on reference scenario. Countries which by 2060 have surpassed the threshold in the reference scenario do not reduce their stocks but instead continue to follow reference scenario trajectories. This approach captures broad empirical patterns without aiming for precise country-level stock-to-wellbeing predictions	Regression based on HDI data (United Nations Development Programme, 2025)
*ShiftToRail*	Modal shift from road to rail vehicle-km with a proportional shift in infrastructure	All regions below the average rail share in total vkm (5.5%) of the top six countries with the highest rail share catch up to this level by 2060, with shares of infrastructure classes per mode kept constant	Own assumption
*MobReduct*	Slowed mobility demand growth	Vehicle-km in 2060 reduced in a steplike manner for income regions: 30% reduction for HI, 20% for UMI, 10% for LMI, no reduction for LI. Reduction in road wear extends road lifetimes proportionally, following the same tiered pattern. Such reductions may be realized through via trip avoidance (e.g., through teleworking and locally available services) and reduced trip distances (e.g., through compact urban design)	Own assumption based on Harvey (2013)
*CarSharing*	Car sharing rate doubled	Parking infrastructure effectively halved by 2060 based on the assumption that car vehicle fleet (two car-owners share one vehicle) and thus demand for capacity is reduced	Own assumption based on Martin et al. (2010)
*FleetElect*	Private passenger fleet electrification	Expansion of charging and stagnation of conventional fueling stations based on complete shift to electric vehicles by 2060	Own assumption
*DemandAll*	Combined demand-side measures	Combined measures of scenarios *ShiftToRail, MobReduct, CarSharing* and* FleetElect*	See above
Supply_*	Combined supply-side measures	Combination with bundle of supply-side measures implemented in MESSAGEix-Materials module: Increased recycling for steel, aluminum, concrete and asphalt, fuel switching, material substitution, and carbon capture and storage (CCS)	Own assumptions implemented in MESSAGEix-Materials (Ünlü et al., 2024)

*SSP2* Shared socioeconomic pathways ‘Middle of the road’ scenario, *vkm* vehicle kilometers, *HI* High-income, *UMI* Upper middle-income, *LMI* Lower middle-income, *LI* Low-incom*e*

Demand-side scenarios represent structural changes in mobility systems and demand. These include convergence of total infrastructure stocks toward a level associated with high human development, modal shifts from road- to rail-based transport, income-specific reductions in overall mobility demand, increased car sharing affecting parking space requirements, and full electrification of the private passenger vehicle fleet implying a shift from conventional fueling stations to e-charging infrastructure. A combined demand-side scenario integrates the individual measures to assess their cumulative effect.

In reality, infrastructure needs may vary with population density and spatial form, with dense urban areas typically requiring less infrastructure per capita than sprawling low-density regions to provide equivalent access (Wiedenhofer et al., 2024). The uniform per-capita target in the StockConv scenario is therefore a deliberate simplification for global exploratory modeling. Density-adjusted benchmarks would likely lower target stocks for densely populated countries, while raise them for sparsely populated ones. Given the highly heterogeneous densities both within and across world regions, a uniform wellbeing indicator-based threshold as used herein provides the most transparent and tractable baseline for cross-country comparison.

Supply-side mitigation options are evaluated through a bundle of circular economy and decarbonization measures implemented in the MESSAGEix-Materials framework. These include material substitution, increased recycling rates for key materials, fuel switching in material production, and the deployment of carbon capture and storage (CCS) technologies in energy-intensive industries. The supply-side measures can be deployed individually, or in combination with economy-wide climate policy, here approximated via cumulative global carbon budget that leads to a greater 67% probability of staying below 2 °C. Note that in the scenarios, CCS is only deployed in combination with climate policy.

Together, these scenarios allow for a systematic assessment of how changes in mobility demand, infrastructure utilization, and material production technologies influence future infrastructure stocks, material requirements, and associated carbon emissions. Individual scenario assumptions are described in more detail in Sect.  3 in Supplementary Information SI1.

### Material flows

Expansion flows are derived from annual net stock changes. Equation 2 summarizes how material inflows *MIF* are calculated. Expansion flows are based on annual stock changes with *MS*_*t-1*_ referring to the stock of the preceding year, while maintenance flows are calculated by dividing the stock in a given year by the infrastructure class-specific lifetime, based on a leaching lifetime modelling approach (van der Voet et al., 2002; Wiedenhofer et al., 2015).

2 $$ MIF_{i,m,t} = \sum \left( {MS_{m,t} - MS_{m,t - 1} } \right) + _{ } \sum \frac{{MS_{i,m,t} }}{{LT_{i, m} }} $$

### Embodied carbon emissions

Calculated annual material demand for mobility infrastructure expansion, maintenance, and replacement is used as input to MESSAGEix-Materials to estimate embodied CO_2_ emissions from material production. End-of-life flows are likewise used to represent material available for re- and downcycling. Emissions are calculated for concrete, asphalt, iron and steel, and aluminum, which account for most emission-intensive infrastructure materials. Emissions from material transport and construction activities, which are estimated to be around 5 to 10% for road construction (Cass & Mukherjee, 2011; Gao et al., 2024), are excluded, as are transportation-related operational emissions. For infrastructure other than roads, it can be assumed that the energy-intensive nature of concrete and steel production would cause even higher shares of material production in total embodied emissions.

## Results

### Current global mobility infrastructure stocks

We estimate global mobility infrastructure stocks at 355 [255–455] Gt in 2024, consistent with previous studies (Rousseau et al., 2022; van Engelenburg et al., 2024; Wiedenhofer et al., 2024). Bulk materials comprising aggregates, asphalt, and concrete dominate these stocks, with aggregates accounting for 74% of total stocks alone (Table 2). Lower-class roads account for the majority of materials (49%), followed by higher-class roads (37%) and rail-based infrastructure (6%). While rail infrastructure represents only a small share of total stocks, it accounts for 68% of steel used in mobility infrastructure, with subway systems comprising 16% of all rail stocks. With less than 3%, all other infrastructure types (parking, fueling and charging infrastructure, airport runways) contribute only marginally.

**Table 2:** Global mean-estimate material stocks in mobility infrastructure in 2024, in million metric tons. ‘High-class roads’ include motorways, primary, secondary, and tertiary roads, while ‘low-class roads’ comprise local and rural roads. ‘Rail-based infrastructure’ includes railway, subway, trams, and associated station buildings, platforms, bridges, and tunnels. More detailed results can be found in Supplementary Information SI2

Material	Higher-class roads (HC)	Lower-class roads (LC)	Roads bridges and tunnels	Rail-based infrastructure	All other infrastructure	Total
Aggregates	96,569	141,748	7,384	12,899	5,599	264,199
Asphalt	31,185	33,404	1,599	0	1,095	67,284
Concrete	3,958	149	6,683	7,414	2,748	20,952
Glass	0	0	0	0	2	2
Iron/steel	0	0	682	1,563	40	2,285
Aluminum	0	0	0	4	< 1	5
Copper	0	0	0	8	< 1	8
Other metals	0	0	0	< 1	3	4
Timber	0	0	0	293	9	301
Plastic	0	0	0	0	< 1	< 1
**Total**	**131,712**	**175,302**	**16,348**	**22,181**	**9,497**	**355,040**

**Fig. 2:**
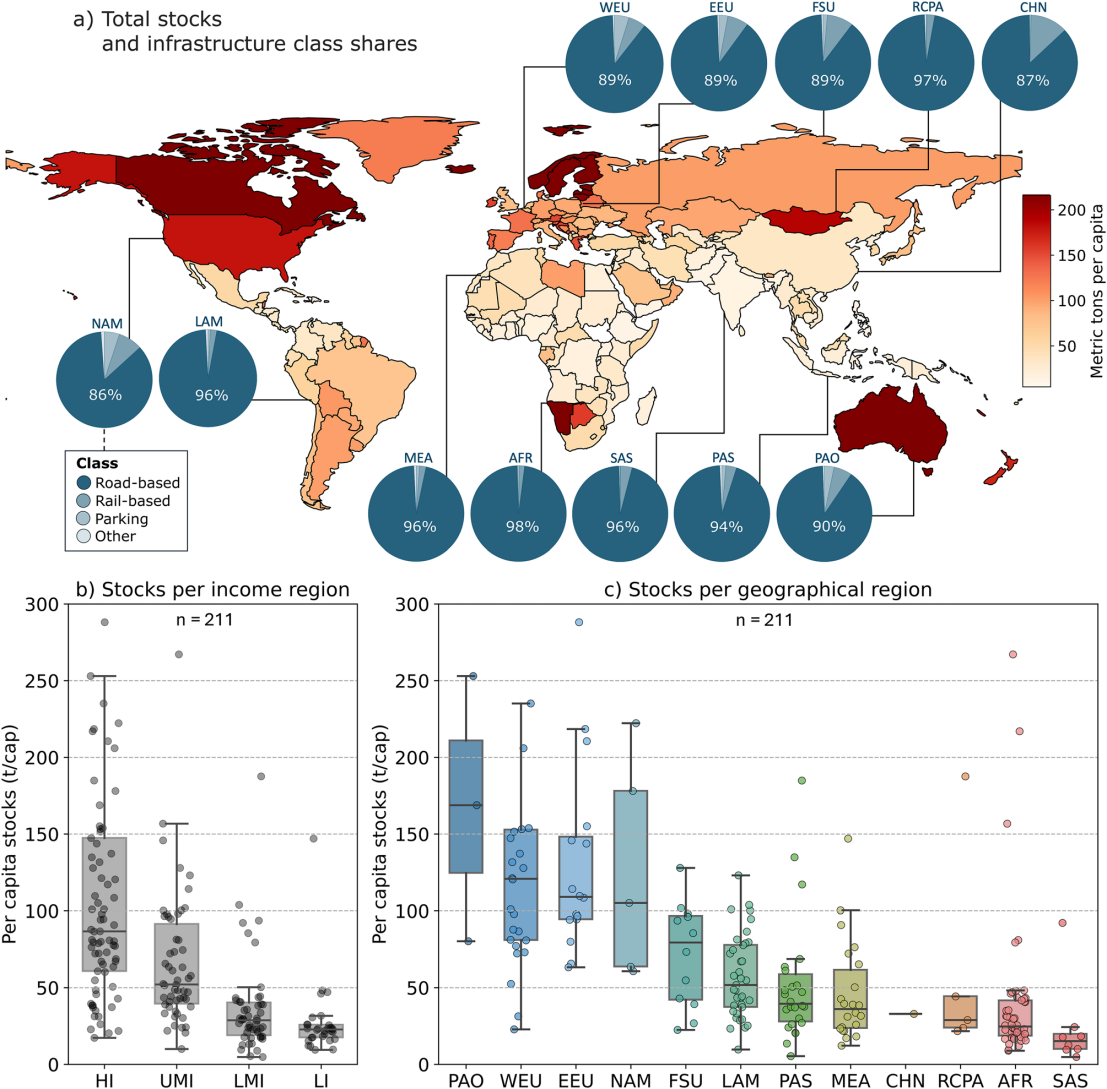
Distribution of global material stocks in mobility infrastructure in 2024. **a** Global map of country-level material stocks in 2024 colored by intensity (per capita stocks) with superimposed pie charts of infrastructure class shares in total stocks per MESSAGEix region. Road- and rail-based infrastructure include associated bridges, tunnels, and station buildings. Other infrastructure includes fueling and charging infrastructure and airport runways. **b** Boxplots of per capita material stocks per income and **c** geographical region, sorted by median (shown as a horizontal line), with boxes representing the interquartile range from the 25th to the 75th percentile of data points. Note that some outliers have been omitted from the figure. *HI* High-income, *UMI* Upper middle-income, *LMI* Lower middle-income, *LI* Low-income, *PAO* Pacific OECD, *WEU* Western Europe, *EEU* Eastern Europe, *NAM* North America, *FSU* Former Soviet Union, *LAM* Latin America and Caribbean, *PAS* Other Pacific Asia, *MEA* Middle East and North Africa, *CHN* China, *RCPA* Rest of centrally-planned Asia, *AFR* Sub-Saharan Africa, *SAS* South Asia. The data for this figure can be found in the Supplementary Information SI2

Mobility infrastructure stocks are highly unevenly distributed globally (Gini = 0.48; Figure S17a in Supplementary Information SI1), with per-capita values ranging from ~ 5 to over 500 t/cap. Figure 2b and c show that low per-capita stocks are found in densely populated, low-income regions such as Sub-Saharan Africa and South Asia, whereas higher stocks are observed in economically developed regions with lower population densities, such as North America and Western Europe. Here, average stock levels are between 100 and 180 t/cap, but were shown to be multiple times higher in some subnational regions (Wiedenhofer et al., 2024), where relative affluence and car dependency are high, while population density is comparably low. Such factors also explain higher per capita stock levels in Southern Africa as compared to the rest of Africa (Fig. 2a).

### Scenarios of mobility stock developments until 2060

**Fig. 3:**
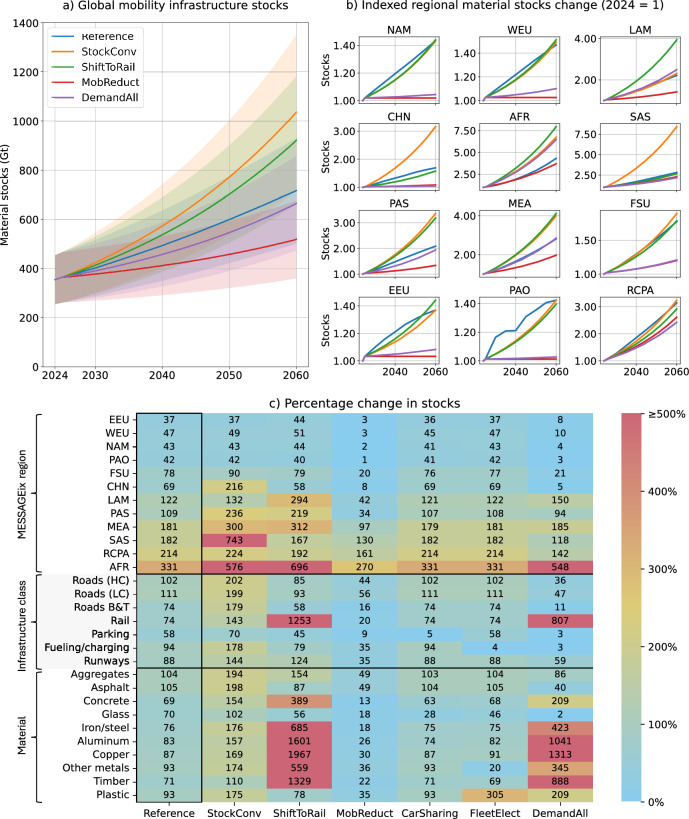
Scenario comparison of change in material stocks and stock composition. **a** Material stock projections for the reference and demand-side scenarios. Uncertainty ranges reflect 25th and 75th percentiles of data points for widths and material intensity factors. **b** Indexed mean-estimate material stock projections for the 12 MESSAGEix regions. For material stocks, scenarios CarSharing and FleetElect are not visibly distinguishable from the reference scenario and consequently not shown. **c** Heatmap of percentage change in mean-estimate stocks relative to 2024 levels per scenario, region, infrastructure class, and material category. *HC* high-class, *LC* low-class, *B&T* bridges and tunnels. The data for this figure can be found in the Supplementary Information SI2

Reference scenario mobility projections suggest an average global increase of 82% in total mobility demand, well in line with other estimates (ITF, 2023). This would require 0.54 million km^2^ of additional infrastructure area, thereby contributing significantly to soil sealing and biodiversity degradation through habitat fragmentation (Shepard et al., 2008). Further, this means a doubling of global mobility infrastructure stocks to 718 [501–932] Gt (Fig. 3a).

In the global stock convergence scenario (StockConv), we see total mean-estimate material stocks triple to 1,037 Gt (Fig. 3), with South Asia and Sub-Saharan Africa seeing the highest percentage increase in stocks, with an 8- and sevenfold increase by 2060, respectively (Fig. 3c). AFR and SAS dominate stock additions in absolute terms, followed by China (Figure S17b in Supplementary Information SI1). Gini coefficients decrease considerably from 0.48 in 2024 to 0.22 in 2060 (see Figure S17a in Supplementary Information SI1) with remaining inequalities attributable to some countries having already surpassed the decent per-capita stock threshold.

With a global shift to rail (ShiftToRail), future additions to road infrastructure as seen in the reference scenario are replaced by the expansion of the railway network. Mean-estimate material stocks increase to 922 Gt in this scenario (Fig. 3a). Latin America (LAM) and Middle East & North Africa (MEA) are main drivers of stock growth, albeit with half of the sevenfold growth in AFR (Fig. 3c).

Under a global, income-tiered mobility reduction (MobReduct), mean-estimate material stocks reach 520 Gt, well below reference scenario levels in 2060 (Fig. 3a).

If the global vehicle fleet was to be halved due to a sharp increase in shared mobility (CarSharing), requirements for future parking space would be reduced. A decarbonisation of the vehicle fleet (FleetElect) increases e-charging infrastructure stocks at the cost of conventional fueling stations. Due to parking space and charging infrastructure making up only a small share of total mobility infrastructure mass, both scenarios result in only very minor changes compared to reference scenario, and are therefore not visible in Fig. 3a.

The DemandAll scenario combines the latter four demand-side scenarios. With 666 Gt in 2060, mean-estimate material stocks are similar to reference levels due to rising infrastructure requirements for rail-based public mobility and mitigating effects of mobility reduction compensating for one another. Absolute and percentage change in total material stocks from 2024 to 2060 per scenario are shown in Fig. 3a and c, respectively. In ShiftToRail, mean-estimate material stocks in rail-based infrastructure increases 14-fold to 300 Gt, with steel, aluminum and copper stocks increasing by a factor 8, 17, and 21, respectively, while timber stocks grow by a factor 14, predominantly due to wood use in railway sleepers. In MobReduct, total stock growth is reduced significantly to 46% compared to the reference scenario. CarSharing and FleetElect both lead to a reduction of less than 1% compared to the reference scenario.

Infrastructure stock growth is heterogenous across world regions (Fig. 3b). Since the decent stock level or the targeted rail share have long been surpassed in higher-income regions, stock growth in StockConv and ShiftToRail here is similar to that of the reference scenario. For other regions, these scenarios increase stocks significantly more than their respective reference scenario, for example in China, AFR and SAS. MobReduct and DemandAll restrict growth of mobility infrastructure significantly, except for regions with strong rail infrastructure expansion.

### Material flows and embodied emissions due to mobility infrastructure

Material stocks are associated with significant annual material flows, both due to stock expansion, as well as maintenance and replacement activities. In the reference scenario, total annual material flows in 2060 amount to 23 [17–29] Gt, with roughly equal shares of maintenance and replacement to expansion activities, with a ratio of 0.8:1 (Figure S15 in Supplementary Information SI1). While 69% of maintenance flows are concentrated in higher-income countries, expansion flows are distributed more evenly across higher- and lower-income countries. In StockConv, annual material flows in 2060 are 46 [34–58] Gt/yr, with 65% used for expansion activities. In ShiftToRail, material flows reach 38 [29–46] Gt/yr, with 66% due to expansion. In comparison, MobReduct requires only 14 [11–18] Gt/yr of material flows in 2060, of which 49% are due to expansion.

**Fig. 4:**
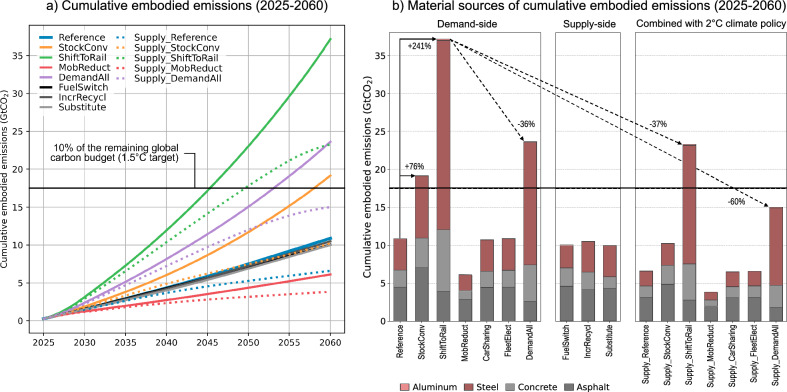
Cumulative embodied CO_2_ emissions from 2024 to 2060. **a** Timeline of cumulative embodied CO_2_ emissions with scenarios that include supply-side measure bundle shown as dotted lines and indicated by the prefix ‘Supply_’. **b** Material composition of cumulative embodied CO_2_ emissions per individual demand-side measures, individual supply-side measures, and combined demand- and supply-side measures with 2 °C climate policy scenario, the latter including carbon capture and storage (CCS). CarSharing and FleetElect scenarios not shown due to negligible variation from reference scenario. Horizontal lines and white bars indicate 10% of the 1.5 °C remaining global carbon budget, corresponding to 1.5% of the 2.0 °C budget. Embodied CO_2_ emissions disaggregated by material and region can be found in Supplemental Information SI2

The GLOMIS-MESSAGEix-Materials model interface (see Figure S2 in Supplementary Information SI1) allows for an assessment of future embodied carbon emissions from material production for infrastructure expansion, maintenance and replacement. Cumulative embodied emissions of mean-estimate material flows for all scenarios are shown in Fig. 4a. From 2024 to 2060, cumulative emissions amount to 11 GtCO_2_ in the reference scenario. In StockConv, cumulative emissions amount to 19 GtCO_2_, an increase of 76%. Most significant reductions can be achieved with reductions in future mobility demand, with cumulative embodied emissions reduced by 44%, to 6 GtCO_2_, in the MobReduct scenario. Other demand-side measures (CarSharing, FleetElect) have negligible emission-saving potentials. This is because these measures are focused on reducing both operational and embodied emissions of the rolling stock which is not considered here, and because associated infrastructure classes only have a low share in total material demand. In the case of ShiftToRail, embodied emissions increase by 241% (37 GtCO_2_) relative to the reference scenario – driven mainly by increased concrete and steel production.

We combined each of the above demand-side measure with a set of supply-side measures (dotted lines in Fig. 4a), namely recycling, material substitution, fuel switching, and CCS. Without more comprehensive climate policy that incentivizes the decarbonization of the entire economy, individual supply-side measures are shown to have a limited mitigation effect, reducing embodied CO_2_ emissions by 4 to 8% relative to the reference scenario (see supply-side bracket in Fig. 4b). However, when combining the reference and StockConv scenarios with supply-side measures and economy-wide climate policy, cumulative emissions decrease by 40% (to 7 GtCO_2_) and 47% (to 10 GtCO_2_) relative to the respective scenarios without supply-side measures and comprehensive climate policy. The mitigation potential of an integrated demand- and supply-side measures scenario (Supply_DemandAll: 15 GtCO_2_, + 38% relative to the reference scenario) is lower than that of MobReduct alone, as emission-intensive production of materials for rail-based infrastructure expansion (see Fig. 4b) offset emission savings through other demand-side measures. Nevertheless, when a modal shift to rail is implemented, highest emission reductions can be achieved through combined demand- and supply-side measures.

Finally, we compare modeled embodied CO_2_ emissions from global mobility infrastructure with the remaining carbon budget for limiting warming to 1.5 °C and 2 °C (Fig. 4b). The reference scenario would lock in 1.0 to 6.2% of the 2 °C and 1.5 °C carbon budgets. Locking in around a quarter of the 1.5 °C budget, an even more substantial fraction would be used in the steel- and concrete-intensive ShiftToRail scenario. In Supply_MobReduct, combining mobility reduction with supply-side measures, most significant reductions can be achieved, reducing the share to 0.3 to 2.2%. These results highlight the potential for infrastructure planning to influence long-term carbon lock-in. See Sect.  2.4 of Supplementary Information SI1 for more detailed results.

## Discussion

### Resource requirements of a ‘great catch-up’ to globally equal infrastructure stock levels

Global mobility infrastructure is expected to undergo significant transformation by 2060, particularly in middle- and lower-income regions. In a reference scenario, the projected growth in vehicle-km drives global material demand and embodied emissions in mobility infrastructure. In a global stock convergence scenario, material demand is amplified by affluence and mobility increases in developing regions. That is not to say that more affluent regions do not also contribute to resource demand and embodied emissions, as shown in Fig. 3c. Due to saturating stocks at a high level, maintenance and replacement requirements are substantial in higher-income regions, and consequently contribute increasingly to global embodied emissions (Figure S15 in Supplementary Information SI1). In contrast, it can be expected that the majority of infrastructure growth will take place in lower-income regions. By 2060, the share of current low and lower middle-income countries in global material stocks in mobility infrastructure will be nearly 50%. This is the case for South Asia and Sub-Saharan Africa (Fig. 3c) where rapid economic development is expected in the near future due to population growth and industrialization (IIASA, 2024b).

Previous studies linked economic prosperity with increased mobility, both for non-occupational personal mobility (Frias-Martinez et al., 2012) and the transport of materials and goods (Listiono, 2018; Sessa & Enei, 2009). Adequate mobility infrastructure is therefore seen as fundamental for economic development (Rietveld & Bruinsma, 2012; Wenz et al., 2020), as accessible transportation networks allow people to commute to workplaces and thereby seizing work opportunities that would otherwise be unavailable to them (Venter et al., 2019). While an increase in mobility and associated material stocks as in the StockConv scenario might seem unrealistic, the rapid accumulation of material stocks in China in the past three decades has shown that with rapid economic development, a large-scale expansion of mobility infrastructure stocks within a relatively small timeframe is feasible. For example, Song et al. (2021) showed that total material stocks in China increased by factor 3.8 between 1990 and 2018. Likewise, road and railway stocks increased from 2 Gt in 1978 to 7 Gt in 2008 (Han & Xiang, 2013). Rapid stock growth is not unique to infrastructure, with floor area in India and Africa expected to grow by a factor 2.2 by 2060 (Ma et al., 2025).

From a sustainability perspective, convergence at a specific per capita stock level is not a meaningful target per se, because per capita stocks are only a poor proxy for the service provisioning and the level of accessibility achieved (Kalt et al., 2019). Importantly, achieving high levels of well-being and decent mobility standards saturate at an intermediate level and only marginal benefits can be expected beyond that (Virág et al., 2022a, 2022b). Furthermore, distance travelled, commonly measured as vehicle-km, is also not a direct or appropriate indicator for the level of mobility service provided, as traveling longer distances is not necessarily more desirable. Rather, reaching all destinations of relevance safely and sustainably is relevant. Likewise, different modes of transportation can provide the same service level. Locally, however, modal choice is often constrained by the availability of infrastructure. This is often the case in rural areas, where facilities (e.g., shops, schools, hospitals) are sparsely distributed and infrastructure is less dense. This reinforces the effect that people living in peripheral areas have to travel longer distances and often use less sustainable modes of transport, such as cars (Dyba & Rotchniak, 2024).

### The role of urban form for future mobility research

The projected material and emission burdens of infrastructure expansion highlight that the key question is not how much infrastructure is built, but what kind and where, making urban form a central determinant of future mobility systems. Economic development is closely tied to urbanization (Bloom et al., 2008) and anticipated economic growth in the Global South – particularly in South Asia and Sub-Saharan Africa (Kundu & Pandey, 2020) – is expected to drive demand for intra- and interurban mobility. Because the urban form established in the present will lock in long-term future mobility patterns and their associated resource use and emissions, the challenge of providing adequate infrastructure also presents an opportunity to construct sustainable infrastructure.

Urban form strongly shapes mobility patterns. Decentralized, car-oriented development reinforces automobile dependence (Brenner et al., 2024a, 2024b), whereas dense, well-planned cities enable more efficient public and active transport systems (Mattioli et al., 2020). Although rail-based public transport infrastructure must be expanded to meet future demand, it is preferable to car-based infrastructure as it can provide equivalent mobility services with substantially lower infrastructure requirements and operational emissions.

Urban mobility accounts for 40% of global passenger transport carbon emissions (International Transport Forum, 2021), making urban mobility infrastructure a key leverage point for climate change mitigation. Empirical evidence from cities such as Vienna (Virág et al., 2022a, 2022b) and scenario analyses for Indian cities (Tiwari et al., 2016) demonstrate that shifts toward public and non-motorized transport can significantly reduce resource use, and embodied and operational emissions.

Globally, urban planning faces two interrelated challenges: steering infrastructure development in rapidly urbanizing regions toward sustainable and equitable transport systems, and reducing car dependence in rural and semi-urban areas by preventing out-migration of services to peripheries (Fertner et al., 2015), and strengthening public and active transport (Thomas et al., 2024). Addressing these issues requires integrated transport and land use planning (Hrelja & Rye, 2023), with concepts like ‘superblocks’ offering potential models for sustainable urban development (Brenner et al., 2024a, 2024b). Such spatial strategies are foundational to the implementation of demand-reducing measures, which are shown here to substantially lower overall resource use. Concretely, urban planning enables a reduction in mobility demand by shaping urban morphologies to minimize travel distances (e.g., through local service provisioning), by providing safe infrastructure for walking and cycling, and by designing transit-oriented development that makes car-free mobility convenient rather than coercive.

### The need for an integrated approach: combining demand- and supply-side measures

To satisfy rising mobility demand, infrastructure can be expanded, shifted, or otherwise modified. At the national level, measures modelled in StockConv and ShiftToRail counteract regional stock disparities (Figure S17 in Supplementary Information SI1), but drive the use of emission-intensive materials such as steel (Fig. 3c, Fig. 4b). The modal shift to rail can still be environmentally beneficial even while raising the embodied emissions, if operational emission savings from vehicle usage are considered (Virág et al., 2022a, 2022b). Such a shift is also socio-economically desirable, as cars may be less accessible to lower-income groups (Venter et al., 2019). Reducing mobility demand has the highest individual potential to reduce not only resource use and embodied emissions but also soil sealing.

Demand-side measures, especially mobility reduction, significantly cut resource use and embodied emissions, far surpassing supply-side measures (Fig. 4b). Nevertheless, while not having the same potential on their own as demand-side measures, supply-side measures such as fuel switching in production, material substitution, increased use of recycled materials, and CCS play an important role in further reducing resource consumption and carbon emissions during the production of materials required for the remaining stock expansion and maintenance. This is particularly important with regard to a shift to rail transport, as lowering operational emissions from private vehicles indirectly increases embodied emissions in public transport infrastructure in a second-order effect. Here, supply-side measures help offset these production impacts.

Neither demand- nor supply-side measures alone suffice to curb resource use from mobility infrastructure expansion. Only an integrated approach was shown to considerably curb cumulative embodied emissions to roughly one third (Fig. 4b) while simultaneously allowing for implied operational emissions to be lowered through fleet electrification and a modal shift to public transportation. Combining both approaches maximizes the mitigation potential, particularly if both embodied and operational emissions are considered.

### Mobility infrastructure and the remaining global carbon budget

Depending on mode and local contexts, embodied emissions of infrastructure expansion and maintenance are estimated to account for up to 30% of overall operational and embodied carbon footprint of modes (Hill et al., 2012). Our findings highlight that future mobility infrastructure expansion and maintenance cause considerable resource demand and embodied emissions. While these are smaller compared to operational emissions, they are far from negligible: depending on the pace of expansion and modal shifts, they can consume several percent of the remaining global carbon budget. Even under global infrastructure convergence and a strong shift towards rail, the combined embodied and operational emissions are expected to take up an increasingly significant share of that budget.

The activity levels used to determine the infrastructure emissions in the baseline scenario are derived from MESSAGEix-Transport. The corresponding cumulative operational emissions from these activity levels from 2020 to 2060 are estimated to be around 300 GtCO_2_ for the transport sector as a whole, excluding aviation. These emissions are comparable to other estimates found in literature. The International Energy Agency (IEA) estimates direct operational emissions of global road- and rail-based transportation to be 6.3 GtCO_2_ in 2023. Using SSP2 ‘middle of the road’ mobility projections as utilized in the reference scenario, the IPCC AR6 scenario database (Byers et al., 2022; Jaramillo et al., 2022) reports cumulative direct operational carbon emissions to reach 260–270 GtCO_2_ until 2060 – nearly 30 times greater than the embodied emissions of infrastructure. However, considering that vehicle operation is further stimulated by infrastructure expansion, growth in the road network would be accompanied by significant increases in distances travelled and consequently operational emissions (Thomas et al., 2024).

These results underscore the need for overall reductions in mobility demand. Further, our findings also imply a trade-off in emissions, as emission-savings from shifting towards decarbonized rail-based transportation are partially offset by embodied emissions from railway infrastructure expansion and associated steel production increases. Consequently, mobility strategies and climate change mitigation policies must carefully weigh these trade-offs – particularly when large-scale infrastructure development is involved. While a shift to rail is essential to reduce car-based mobility and at the same time enables access mobility services for all, prioritizing other forms of structural change in mobility provisioning are likely to yield even higher emission reductions. Replacing motorized with active mobility, particularly in dense, urban settings, would require comparably few infrastructure investments beyond improving safe walking or cycling infrastructure, and would also have co-benefits for public health by increasing physical activity and improving air quality.

Our analysis focuses on embodied emissions related to mobility infrastructure. Embodied emissions of the vehicle fleet and vehicle operational emissions are thus beyond the scope of this study. However, we stress that an integrated framework jointly modelling infrastructure systems and vehicle stock turnover and operation remains a critical area for future research.

## Conclusions

This study demonstrates that the projected global expansion of mobility infrastructure, essential for economic development and equitable access, poses a challenge to resource efficiency and climate goals. Our exploratory scenarios show that without intervention, infrastructure stocks could more than double by 2060, consuming a substantial share of the remaining carbon budget through embodied emissions. Further, we highlight a critical tension between operational benefits of rail expansion and its high embodied emissions from material use.

The findings underscore that demand-side measures, particularly reducing overall mobility demand, are the most effective lever for curbing both material use and embodied emissions. However, neither demand- nor supply-side strategies alone are sufficient. An integrated approach has the highest potential to cut embodied emissions of infrastructure expansion while providing adequate global mobility services, helping to offset the embodied carbon cost of expanding low-operational-emission transit systems.

Ultimately, the question is not merely how much infrastructure to build, but what kind and where. The urban form established today will lock in long-term mobility patterns. Therefore, coordinating transport and land-use planning is key to supporting demand-side measures, preventing long-term infrastructure and carbon lock-ins, and steering infrastructure development toward sustainable transport systems.

## Summaries:

### Supplementary Information SI1:

Provides detailed descriptions of the methodology, including model assumptions, scenario narratives, and input data. Further, it provides information on model uncertainties and limitations, as well as additional results.

### Supplementary Information SI2:

Provides all data that was used in the figures of the main article. Additional disaggregated data is provided, as is a country code correspondence table that facilitates the interpretation of figures and data.

## Supplementary Information

Below is the link to the electronic supplementary material.

Supplementary file1 (PDF 3942 KB) Supporting information SI1 provides detailed descriptions of the methodology, including model assumptions, scenario narratives, and input data. Further, it provides information on model uncertainties and limitations, as well as additional results.

Supplementary file2 (XLSX 6842 KB) Supporting information SI2 provides all data that was used in the figures of the main article. Additional disaggregated data is provided, as is a country code correspondence table that facilitates the interpretation of figures and data.

## Data Availability

The data that supports the findings of this study are available in the supporting information of this article.
