# Supplementary material for: Material and carbon implications of future mobility infrastructure expansion around the world
Source: J Ind Ecol. 2026 Jul 18;30(4):1935–49. doi: 10.1007/s44498-026-00132-x (PMC13451253; doi:10.1007/s44498-026-00132-x)
Supplement: Supplementary file 1 [file 44498_2026_132_MOESM1_ESM.pdf]

SUPPORTING INFORMATION FOR:

Baumgart, A., Ünlü, G., Grammer, B., Javaid, A., Maczek, F., Krausmann, F., Krey, V. & Wiedenhofer, D. Material and carbon implications of future mobility infrastructure expansion around the world. *Journal of Industrial Ecology.*

Summary

The supporting information provides detailed descriptions of the methodology, including model assumptions, scenario narratives, and input data. Further, it provides information on model uncertainties and limitations, as well as additional results.

Supporting information text

1. Methodology and data

This study aims to model global material stocks and flows of mobility infrastructure and associated embodied CO_2 emissions. Scenario narratives are developed and different future stock trajectories are modelled. Both demand- and supply-side measures are evaluated against the backdrop of the remaining global carbon budget.

1.1. Scope and system definition

GLOMIS (GLObal Mobility Infrastructure Stocks) is a dynamic stock-driven material flow analysis (MFA) model developed in Python to estimate materials stocks and flows. The model is integrated with the MESSAGEix-Materials (Ünlü et al., 2024) integrated assessment model to calculate embodied CO_2 emissions with annual material in- and outflows serving as soft-links between the two model spheres.

The GLOMIS model is global in scope, but generates country-level results which are then analyzed at the world-regional level, using both the 12-regional resolution of the MESSAGEix model, as seen in Figure S1, and the four World Bank income levels (World Bank, 2024). Open-access, crowd-sourced data is used to quantify stocks for the year 2024. Stocks are then projected to the year 2060, covering more than three decades of stock development. Any further projection was deemed too hypothetical considering possible changes in infrastructure expansion and limitation policies.

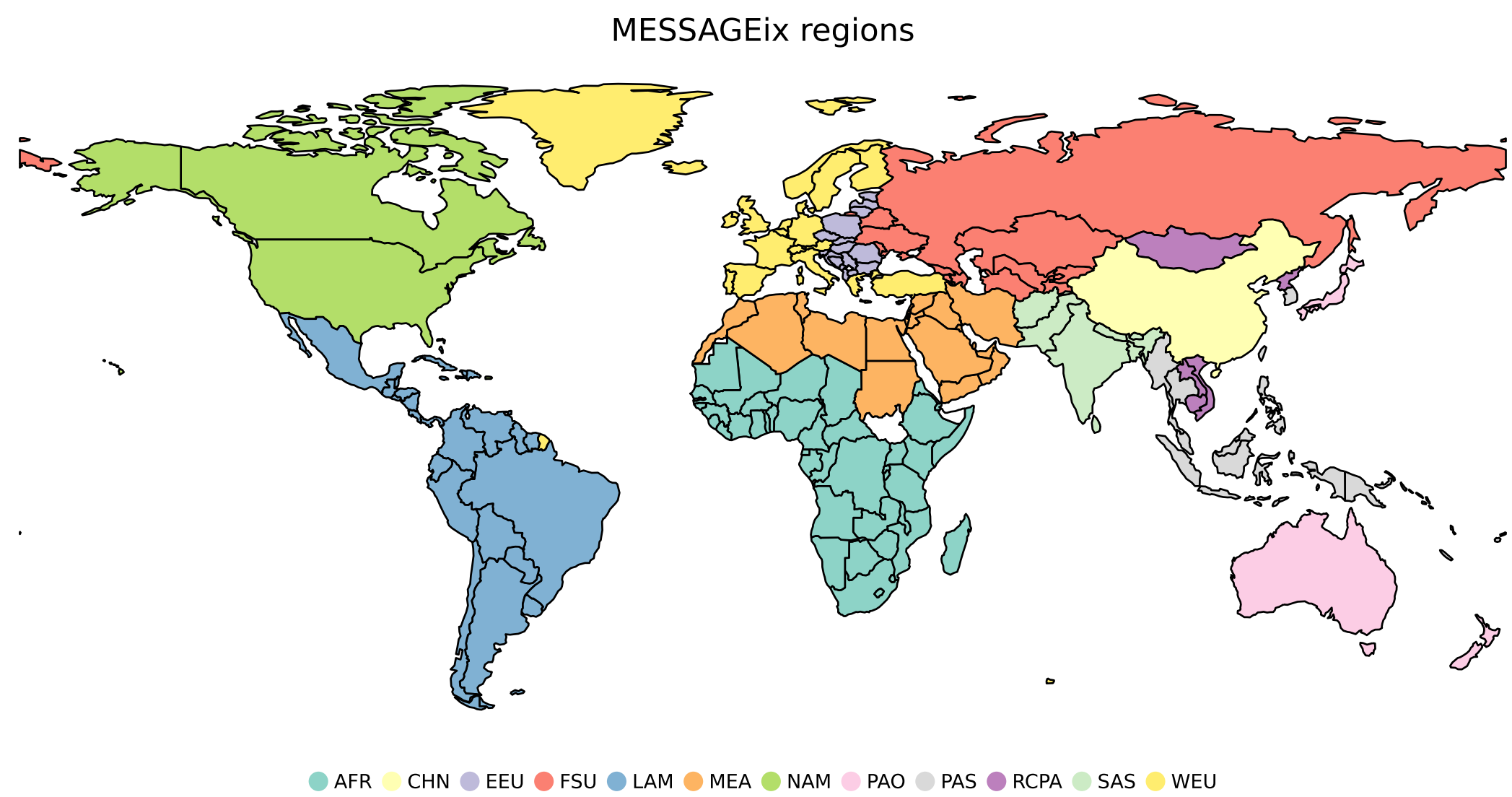

Fig. S1. MESSAGEix regions as used in this study. PAO = Pacific OECD, WEU = Western Europe, EEU = Eastern Europe, NAM = North America, FSU = Former Soviet Union, LAM = Latin America and Caribbean, PAS = Other Pacific Asia, MEA = Middle East and North Africa, CHN = China, RCPA = Rest of centrally planned Asia, AFR = Sub-Saharan Africa, SAS = South Asia.

Ten different materials are covered: Iron/steel, aluminum, copper, other metals (a mix class of various metals occurring in small amounts, often not classified consistently in literature), aggregates (sand and gravel, excluding aggregates embodied in asphalt and concrete), concrete, glass, asphalt, plastic, and timber. In total, 37 different infrastructure classes are differentiated. These include various road classes, rail-based infrastructure, airport runways, and bridges and tunnels. GLOMIS expands the scope of the predecessor studies by also including parking infrastructure (parking lots, building, and garages), railway and subway station buildings as well as platforms, and fueling and charging infrastructure. A detailed list of infrastructure classes, as well as the respective OSM tags they are compiled from, can be found in supplementary information.

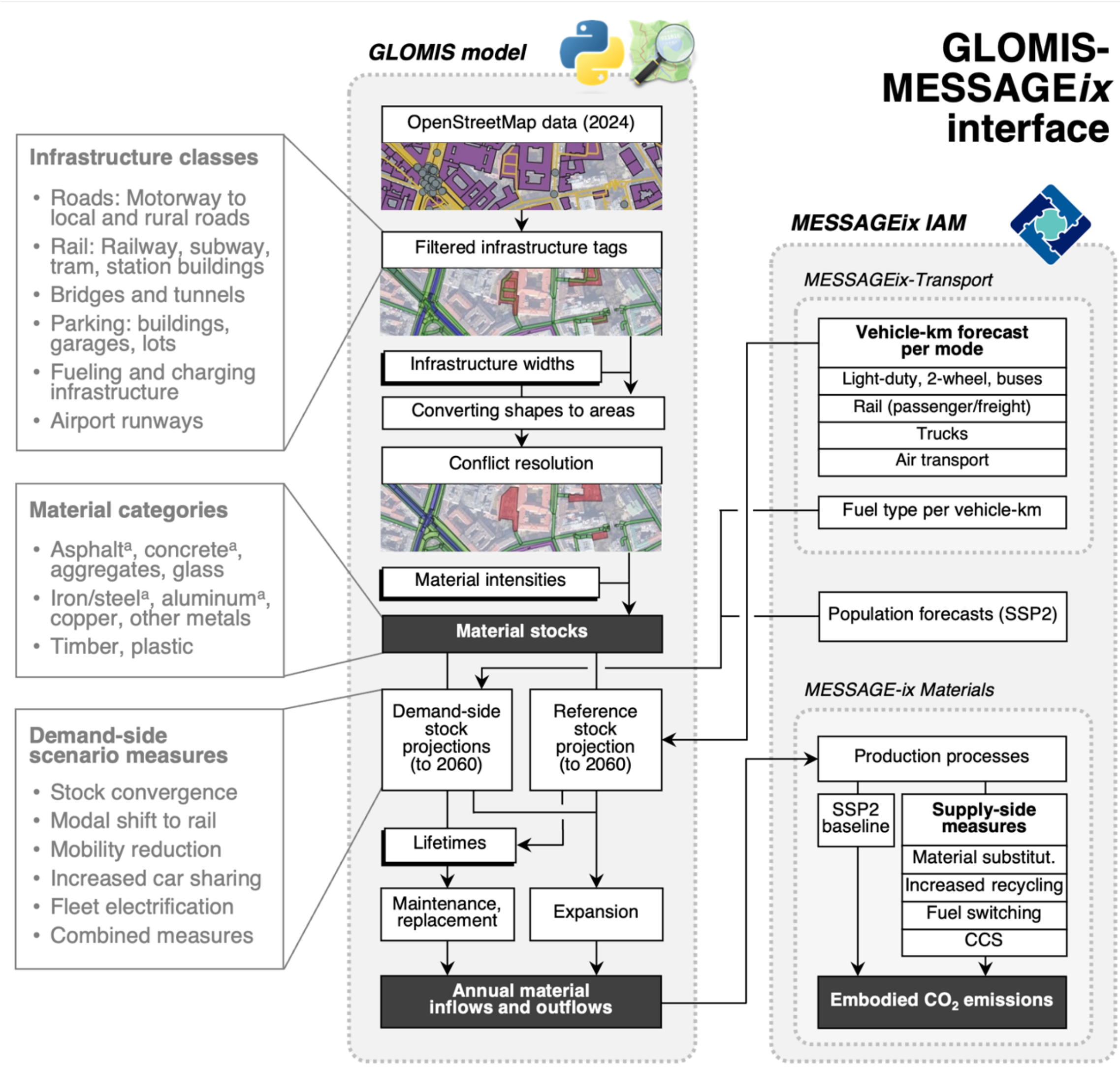

Fig. S2. Model framework showing GLOMIS and MESSAGEix model processes and interlinkages. Model scope encompassing infrastructure classes, material categories, and demand-side scenario measures shown in the left column. Abbreviations: LDV = Light-duty vehicle, CCS = carbon capture and storage, SSP2 = Shared socio-economic pathway 'Middle-of-the-road' scenario.
[a] Materials for which embodied CO_2 emissions are quantified in MESSAGEix-Materials.

The GLOMIS-MESSAGEix interface (see Figure S2) covers material gross additions to stocks (GAS), material flows for maintenance and replacement of stocks, and end-of-life (EOL) demolition and recycling flows, as well as embodied CO_2 emissions, i.e., emissions occurring during the production phase. Emissions occurring during extraction, transportation, and on-site during construction or assembly, operation and demolition are not modelled.

1.2. Modelling material stocks and flows in mobility infrastructure

We designed a workflow to classify and process geographical features from crowd-sourced, publicly available data to map mobility infrastructure stocks. The workflow was exclusively written in Python, with additional error checking and visualization conducted in QGIS.

1.2.1. OpenStreetMap data coverage

To this end we primarily use data from OpenStreetMap (OSM), a collaborative project that provides free and editable maps of the world. OSM data includes various types of geographical features such as points (e.g., EV charging stations), lines (e.g., roads), and polygons (e.g., parking spaces). The data is unstructured in that each feature may possess one or more key-value pairs providing additional information. The use of tags and their values is not enforced, but usually adheres to OSM conventions that allow to classify the data according to its properties.
OSM data is available globally, but since it relies on users to add and update features, coverage and accuracy can vary substantially. North America and Europe have very active OSM communities, while Africa and the Middle East do not[1]. The data was downloaded in the course of August-September 2024 from the Geofabrik, BBBike and OSMfr mirrors.[2]
To process and tile the data, the workflow uses the Global Administrative Boundaries (GADM) dataset version 4.1, released in July 2022[3]. The GADM dataset is compiled from various sources and includes territorial boundaries for 263 countries, autonomous regions and disputed territories. GADM distinguishes between countries and their autonomous regions at the top-level, consequently entities such as overseas departments (e.g., French Guiana) are not included in the national boundaries of France and the respective country statistics.

1.2.2. Workflow: From OpenStreetMap data to infrastructure areas

The workflow iterates over all administrative boundaries provided by the GADM dataset and processes data excerpts at a subdivision level, depending on availability and the boundaries size. This ensures that the processed data tiles are of roughly equal size, fit into the main memory, and can be processed in parallel. The QuackOSM library[4] was used to retrieve the raw data from the OSM mirrors

[1] https://osmstats.neis-one.org/?item=countries
[2] https://download.geofabrik.de, https://extract.bbbike.org, https://tile.openstreetmap.fr
[3] https://gadm.org
[4] https://kraina-ai.github.io/quackosm

(Figure S3a) and bring it into a Python compatible GeoParquet format. Initially, all features are pulled that posses a tag of any value that may be used in the classification. The features are classified into a specific category if their tags match the user provided scheme (see Table S2), while unclassified features are discarded.

Once classified, all features are transformed into polygons (Figure S3b). Line and point features, such as roads, rails, are buffered with a specific width to model the surface areas they occupy (see Table S1 for original shape types per infrastructure class). This provides a fairly accurate spatial representation of line features, while for point features this is necessarily a very crude approximation of their extent. To indicate this, point feature areas are represented as circles. The workflow differentiates assumptions about surface areas and widths by country, to account for local differences in construction. If no country specific values are set, global defaults are used.

This creates explicit spatial extents for all features but introduces overlaps between the features that need to be removed. Overlaps exist primarily within each category, e.g., when local roads intersect at junctions, but can also exist between different feature classes that are mutually exclusive. The vast majority of these are overlaps between roads and parking surfaces, which are often mapped on top of each other in OSM data. Resolving these conflicts (Figure S3c) is computationally expensive, but improves overall accuracy of the surface estimates.

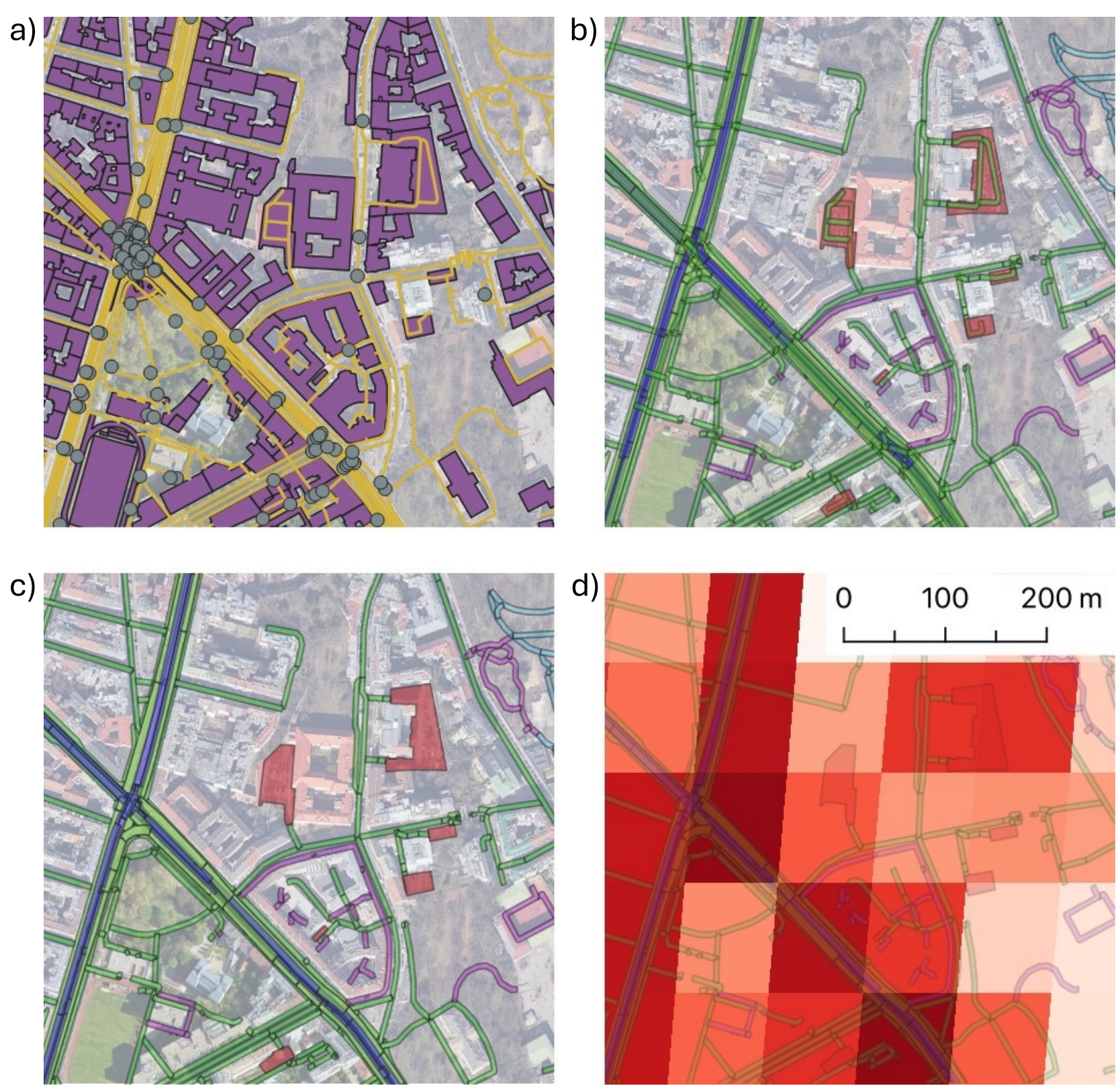

Fig. S3. a) OpenStreetMap raw data; b) Classified infrastructure with modelled areas; c) Classified data after conflict resolution; d) Stock raster after multiplying feature area with material intensities.

Based on combinations of OSM tags, 32 unique infrastructure classes were produced. The main tags used were highway and railway, each denoting the hierarchical class or more precise type of an infrastructure class. For roads, a Geofabrik product description (Ramm, 2022) provided guidance for matching OSM tags with road classes (‘motorway’ to ‘rural roads’) and based on manual samples from Google Street View for various regions, as well as by consulting the OSM online encyclopedia entry for each tag (OpenStreetMap, 2024), each OSM tag was matched with one of the 32 infrastructure classes based on characteristics such as road width, pavement type, etc. While most infrastructure classes are made up of only one or two OSM tags, ‘local roads’ are compilations of multiple road types spanning a variety of functions that broadly share a common width and pavement type. Analogously, rail-based infrastructure classes were matched with the rail infrastructure classes ‘railway’, ‘subway’, ‘tram’, and ‘other rail’.

Table S1. Overview of OpenStreetMap tags, their allocation to infrastructure classes used herein.

**Main category**	**Infrastructure class**	**OpenStreetMap tags**	**Original shape type**
Roads	Motorways	highway=motorway	line
		highway=motorway_link	
	Primary roads	highway=trunk	
		highway=trunk_link	
	Secondary roads	highway=primary	
		highway=primary_link	
	Tertiary roads	highway=secondary	
		highway=secondary_link	
	Local roads	highway=tertiary	
		highway=tertiary_link	
		highway=service	
		highway=residential	
		highway=living_street	
		highway=pedestrian	
		highway=footway	
		highway=cycleway	
		highway=unclassified	
		highway=unknown	
	Rural roads	highway=track	
Road infrastructure	Motorway bridge/tunnel	Same as ‘Roads’ category with tags: bridge=yes / tunnel=yes	
	Primary road bridge/tunnel		
	Secondary road bridge/tunnel		
	Tertiary road bridge/tunnel		
	Local road bridge/tunnel		
	Rural road bridge/tunnel		
Rail	Railway	railway=rail	
	Railway	railway=light_rail	
	Subway	railway=subway	
	Tram	railway=tram	
	Rail (other)	railway=funicular	
		railway=miniature	
		railway=monorail	
		railway=narrow_gauge	
		railway=rack	
Rail infrastructure	Railway bridge/tunnel	Same as ‘rail’ category with tags: bridge=yes / tunnel=yes	
	Subway bridge/tunnel		
	Tram bridge/tunnel		
	Rail (other) bridge/tunnel		

	Subway building	railway/public_transport=station & station=subway	point
	Railway building	railway=station/halt; public_transport=stop_position & train=yes	
	Railway building	building=train_station & train=*	polygon
	Railway building platform	train=yes & railway/public_transport=platform	
Parking	Parking surface	amenity=parking & parking=lane/street_side/surface	
	Parking building	amenity=parking & parking=multi-storey	
	Parking underground	amenity=parking & parking=underground	
	Parking garage	building=garage/garages & amenity=* & parking=*	
Fueling and charging infrastructure	Gas station	amenity=fuel	
	Gas station	amenity=fuel	point
	Gas station building	amenity=fuel & building=*	polygon
	Gas station building	amenity=fuel & building=*	point
	Charging point	amenity=charging_station	

For each road and rail infrastructure class, associated bridges and tunnels were distinguished using the additional OSM tag ‘bridge=yes’ or ‘tunnel=yes’, respectively. In the case of subway lines, the latter refers to underground sections, while the former or the lack of either tag refers to above-ground, elevated sections, respectively. Additional infrastructure classes include railway or subway station infrastructure, parking infrastructure (parking lots and buildings, as well as underground garages), as well as fueling infrastructure (conventional gas stations and electric charging points). These infrastructure classes predominantly make use of the OSM tags ‘amenity’, ‘building’, ‘parking’, and ‘public_transport’.

1.2.3. Error handling

The workflow involves repeated geometry operations and relies on large amounts of unstructured, user-generated data. At various stages, invalid geometries and tag values must be expected and, if possible, repaired. Some errors such as invalid self-intersecting polygons, can often be repaired automatically. Others, such as misspellings in tags or erroneous double classifications (e.g., lines tagged as both road and rail) would require manual correction. Due to the scope of the project and the large number of features involved, this is not feasible. If automated repairs fail, erroneous data is therefore discarded. Discarded data is logged and retrievable from the workflow to ensure this does not impact the overall result.

To resolve some of the conflicts stemming from class overlaps, we defined a set of conflict resolutions. While some infrastructure class overlaps may be realistic (e.g., bridges above or tunnels below roads and railway lines), other overlaps are less reasonable. This is especially true for overlaps of polygons and lines. In the raw OSM data, roads often run through parking infrastructure areas. This is likely due to the use of OSM maps for navigational purposes. However, in reality, it is more likely that a parking lot is made up of a single pavement type. In cases of such overlaps, our conflict resolutions allow the model to select one infrastructure class over the other based on a predefined order: In the case of roads and parking lots (surface parking), motorways, primary and secondary roads are to be selected over parking lots, as it is less likely, that these major road types are interrupted by parking infrastructure, and more likely, the parking infrastructure is located to both

sides of these roads. For tertiary, local and rural roads, however, the opposite is assumed and the conflict is set to be resolved to parking infrastructure. All other infrastructure classes follow the former rule (i.e., parking lots are to be prioritized). The same logic is used for the paved area of gas stations. All building types (gas station buildings, railway station buildings and platforms, as well as subway stations) overwrite all other infrastructure classes (i.e., overlapping road or rail sections are removed). The conflict of overlapping road segments is resolved by always prioritizing the hierarchically higher class (e.g., motorways over primary roads, tertiary over rural roads).
In some cases, such as buildings, additional OSM information were used such as the number of floors. In that case, the area is multiplied by the number of floors. If, however, the floor number of a building is not available, an average number of floors is assumed. Consequently, for parking buildings an average of three floors is assumed, while for underground parking garages, an average of two floors is assumed. For all other building types (gas station buildings, station buildings), an average of one floor is assumed. These likely conservative assumptions are supposed to counterbalance the expected bias in tag use with structures with more floors being more likely to be tagged with information regarding the actual number of floors.

1.2.4. Output product

The primary output of the workflow is the vector data of classified mobility infrastructure. From these, stocks can be calculated by multiplying the material intensity per area unit with their surface areas (Figure S3d). For buildings, such as railway stations or parking garages, the area is multiplied by the number of building levels, if given by the OSM data. Otherwise, default values are used.
Summary statistics can be directly calculated for the administrative boundaries that were used to originally tile the data, but these are not heterogenous due to both data availability and differences in administrative structure for the various countries. For a spatially explicit representation of stocks, a raster product is generated from the vector geometries. The raster images are generated by burning the vector geometries material intensity per area on 2x2 m grid cells, which can then be coarsened to an arbitrary, user-defined resolution.
The rasterization of vector data onto a finite gridded space necessarily introduces some very small divergence between the area covered by the polygon features and their raster representations. To equalize the total stock sums, the resulting difference in mass is distributed or removed evenly from each raster tile.

1.2.5. Deriving material stocks via material intensity factors

Material intensity factors

Material intensity (MI) factors with the unit kg/m^2 were used to derive material stocks for each square meter of mobility infrastructure. The factors were primarily compiled for a predecessor study (Wiedenhofer, Baumgart, et al., 2024), with an additional literature review having been conducted for updating MI factors for

infrastructure classes present in the predecessor study, and for adding new MI factors for infrastructure classes introduced herein (parking and charging infrastructure, station buildings).
While many studies and reports describing the material composition or structure of mobility infrastructure, often these are unusable for our use case. This is because, for example, they define material composition of roads based on either traffic volume or pavement type and not on a hierarchical class from motorways to rural roads. Consequently, only studies were used that explicitly provide material composition information for individual road classes as defined in Table S1.

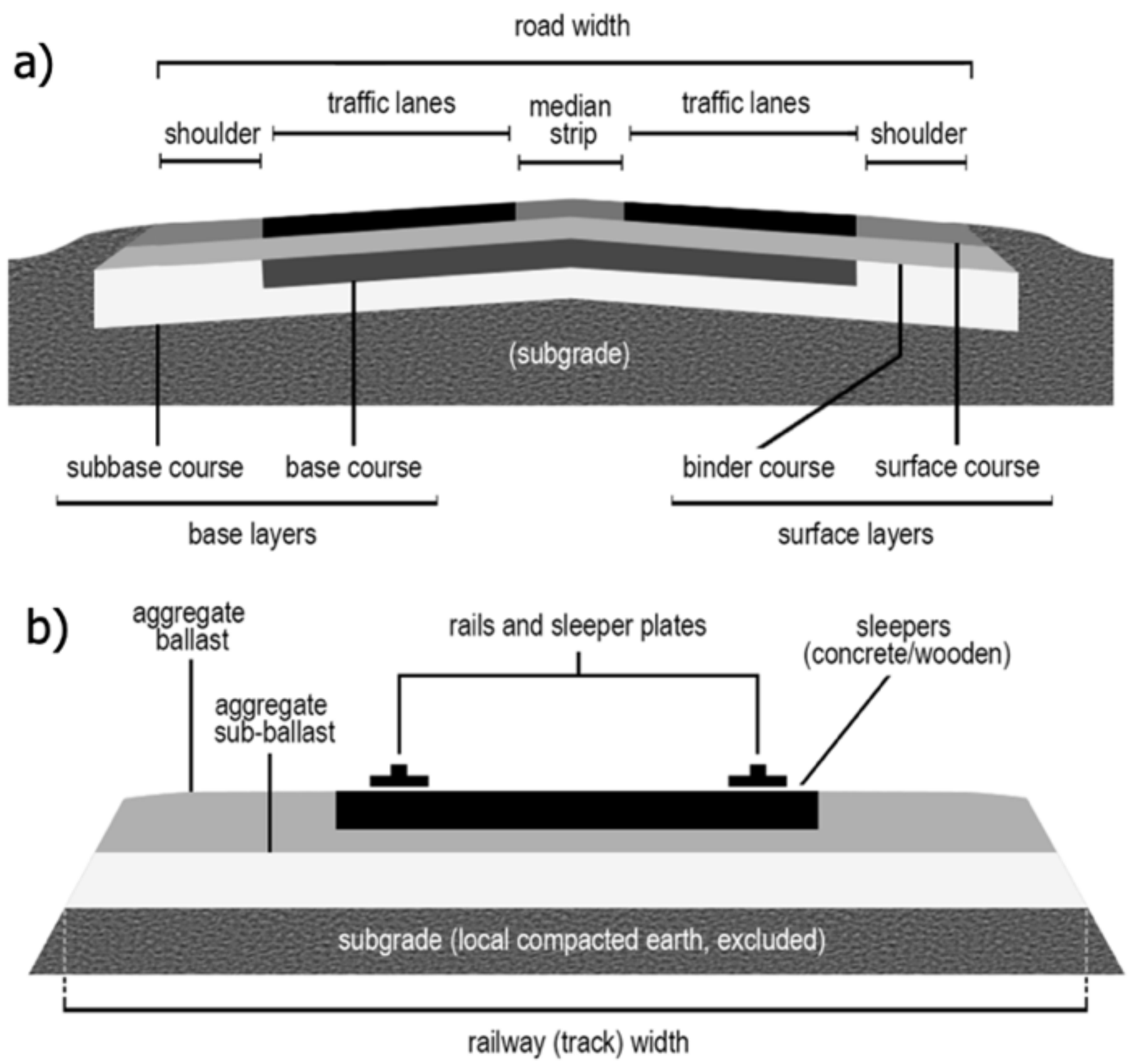

Fig. S4. Simplified road (a) and railway (b) cross section elements used for defining widths and deriving material intensities.

Figures S4a and S4b show the cross sections of roads and railways, respectively. For our study, MI factors were developed for all layers except for subgrade, which is assumed to be local compacted earth that represents material that was not transported or transformed in any meaningful way and was therefore excluded from our study scope. MI factors and widths used in this study, disaggregated by infrastructure class and main material category, can be found in Table S2.

Table S2. Global mean-estimate material intensity factors. Note that categories may not sum to total due to rounding.

Infrastructure class	Material intensity (t/m²)					Width (m)	Data sources
	Biomass	Metals	Minerals	Fossil	Total		
Motorways	–	–	1.76	0.02	**1.79**	22.6	(Alzaim et al., 2020; Alzard et al., 2019; Augiseau & Kim, 2021; Chen et al., 2017; Cruz, 2016; CSIR, 2000; DMR, 2014; DMT, 2016; Frantz et al., 2023; Haberl et al., 2021; Henderson & van Zyl, 2017; Lanau & Liu, 2020; Miatto et al., 2021; Özgenel, 2016; Wiedenhofer, Baumgart, et al., 2024; Wiedenhofer, Schug, et al., 2024)
Primary roads	–	–	1.44	0.02	**1.46**	15.0	
Secondary roads	–	–	1.27	0.02	**1.29**	11.6	
Tertiary roads	–	–	1.07	0.01	**1.08**	9.2	
Local roads	–	–	0.56	0.01	**0.56**	5.7	
Rural roads	–	–	0.29	<0.01	**0.29**	5.0	
Motorway bridges	–	0.14	3.17	0.02	**3.33**	21.5	(JICA, 2018; Haberl et al., 2021; Wiedenhofer, Baumgart, et al., 2024)
Primary bridges	–	0.16	2.64	0.02	**2.81**	11.7	
Secondary bridges	–	0.16	2.47	0.1	**2.64**	10.9	
Tertiary bridges	–	0.16	2.30	0.01	**2.47**	9.1	
Local bridges	–	0.16	2.13	0.01	**2.30**	4.7	
Rural bridges	–	0.16	2.13	<0.01	**2.30**	4.7	
Motorway tunnels	–	0.12	5.14	0.02	**5.29**	21.5	
Primary tunnels	–	0.12	4.79	0.02	**4.93**	11.7	
Secondary tunnels	–	0.12	4.63	0.1	**4.77**	10.9	
Tertiary tunnels	–	0.12	4.43	0.01	**4.57**	9.1	
Local tunnels	–	0.12	4.25	0.01	**4.38**	4.7	
Rural tunnels	–	0.12	4.25	<0.01	**4.38**	4.7	
Railway	0.01	0.02	0.45	–	**0.48**	11.0	(Antoniou et al., 2023; Bai et al., 2019; Frantz et al., 2023; Haberl et al., 2021; Schmied & Mottschall, 2013; Wiedenhofer, Baumgart, et al., 2024)
Railway bridge	0.01	1.14	0.86	–	**2.00**	10.8	
Railway tunnel	0.01	1.14	4.49	–	**4.64**	10.8	
Subway. ground	–	0.20	2.27	–	**2.46**	10.0	
Subway, elevated	–	0.28	3.98	–	**4.25**	10.0	
Subway, underground	–	0.55	10.56	–	**11.20**	10.0	
Tram and other rail	0.01	0.02	0.45	–	**0.47**	6.3	
Railway station	–	–	4.40	–	**4.40**	[1]500.0	
Railway platform	–	–	4.80	–	**4.80**	–	
Subway station	–	–	6.05	–	**6.05**	[1]1.500.0	
Gas station	–	0.28	0.06	–	**0.34**	[1]1.000,0	(Haberl et al., 2024; Lucas et al., 2012; Mulrow & Grubert, 2023; Zhang et al., 2019)
Gas station buildings[2]	0.04	0.05	1.03	<0.01	**1.12**	[1]500.0	
Charging station	–	0.17	0.82	0.07	**1.06**	[1]1.0	
Parking, surface	–	–	0.72	0.01	**0.73**	[1]1.000,0	(Cruz, 2016; Eliassen et al., 2019; Rebello, 2022; Zeitz et al., 2019)
Parking building	–	0.05	2.52	–	**2.56**	[1]1.000,0	
Parking, underground	–	0.01	1.25	–	**1.25**	[1]1.000,0	
Airport runway (rigid)	–	–	1.06	–	**1.06**	–	(Wiedenhofer, Baumgart, et al., 2024)
Airport runways (flexible)	–	–	0.96	0.01	**0.97**	–	

[1] Width refers to assumed area in m² if infrastructure is provided by OpenStreetMap as points instead of polygons.
[2] General commercial building MI factors have been used as proxies for fuel station buildings.

The scope of the model is global. Country-specific data was used for Germany, Austria, Slovakia (Haberl et al., 2021), France (Augiseau & Kim, 2021), Denmark (Lanau & Liu, 2020), and Sweden (Cruz, 2016). For countries or individual infrastructure classes where no country-specific MI or width data points were available, global averages across all regions were used that also include data from non-EU27 countries including the United Kingdom, United States of America (USA), China, Japan, Nepal, Turkey, United Arab Emirates, and South Africa. Only roads for the USA and China were not considered in global averages due to regional specific characteristics such as a relatively high share of concrete (rigid) pavements in roads.

Local and rural roads are quite heterogenous infrastructure classes with multiple function types for local roads (service roads, footways, cycleways) and multiple track grade types for rural roads (OSM tag grade=1-5). Based on our manual samples in Google Street View, we find that local and rural roads (tracks) vary in pavement type across specific road types and grades. Consequently, the rural road MI is weighted at the national level based on shares of each OSM track grade's length in the total length of tracks. While track grade types 1 is assumed to be asphalted, the remaining track grades are assumed to be 50% gravel roads and 50% so-called dirt roads (i.e., merely consisting of compacted local earth or other local materials). Because for dirt roads no material is extracted, transported, or otherwise moved, they do not fall within our socio-metabolic system boundary and their MI factor is effectively zero. Track roads without a grade information tag are weighted according on the shares of the reported grade types per region. For local roads, we assume that 50% are paved and 50% are unpaved with 75% of unpaved local roads being compacted local material (MI = 0). For further information on how MI factors were derived, refer to Wiedenhofer et al. (2024).

Calculating material stocks and flows

As a stock-driven flow model, GLOMIS first computes the material stock for the baseline year 2024. Equation 1 was used to calculate material stocks *MS* for the base year, with *A* referring to the area per infrastructure class *i* in year *t*, *W* refers to the infrastructure width, and *MI* to the material intensity per material *m*.

$$MS_{m,t} = \sum_{i} (A_{i,t} \times MI_{i,m}) \qquad \text{Equ. 1}$$

In a reference scenario, material stocks are projected to the year 2060 using mobility data (vehicle-km) for 12 different regions and modes as proxy. Material inflows consist of expansion, maintenance and replacement flows. Equation 2 summarizes how material inflows *MIF* are calculated. Expansion flows are based on annual stock changes with MS_{t-1} referring to the stock of the preceding year, while maintenance flows are calculated by dividing the stock in a given year by the infrastructure class-specific lifetime, based on a leaching lifetime modelling approach (van der Voet et al., 2002; Wiedenhofer et al., 2015). Table S3 provides an overview of global lifetime assumptions. Maintenance inflows replace material removed from stock. Because infrastructure stocks are assumed to not shrink in

absolute terms, as they rather enter hibernation in case of disuse, maintenance flows equal material outflows.

$$MIF_{i,m,t} = \sum \left(MS_{m,t} - MS_{m,t-1} \right) + \sum \frac{MS_{i,m,t}}{LT_{i,m}} \quad \text{Equ. 2}$$

To account for an assumed time delay in infrastructure expansion (e.g., due to a gap between legislature and its implementation), an exponential interpolation was used, thereby accounting for a start-up phase.

Total annual material demand quantified in GLOMIS is used as input for MESSAGEix-Materials where CO_2 emission occurring during the production phase are calculated. Likewise, total outflows represent material available for re- and downcycling in MESSAGEix-Materials.

Table S3. Lifetime assumptions in years per infrastructure class and material, as used in this study. Values based on those used in Wiedenhofer et al. (2024), except for fueling and charging stations, parking, and railway stations (own assumptions). B&T = bridges and tunnels.

Material class	Roads	Road B&T	Railway	Railway B&T	Subway	Tram/ other	Fueling/ charging	Parking (surface)	Parking (other)	Railway stations	Run-ways
Iron/steel	–	120	30	120	120	30	30	–	80	–	–
Copper	–	–	50	120	120	50	30	–	–	–	–
Aluminum	–	–	50	120	120	–	30	–	80	–	–
Other metals	–	–	50	120	–	–	30	–	–	–	–
Concrete	33	120	50	120	120	50	30	33	80	120	33
Asphalt	29	29	–	–	–	–	29	29	–	–	29
Aggregates	120	120	120	120	120	120	120	120	–	120	120
Glass	–	–	–	–	–	–	20	–	20	–	–
Timber	–	–	50	50	–	–	20	–	–	–	–
Plastic	–	–	–	–	–	–	20	–	–	–	–

1.3. Scenario design: demand- and supply-side measures

Demand-side prospective scenarios are developed based on mobility, a decent stock level threshold and other assumptions, as outlined in Table S4. In total, there are demand-side scenarios that determine future stock levels and annual materials flows of mobility infrastructure expansion, maintenance, and replacement.

When calculating emissions, the above demand-side scenarios are combined with a bundle of various supply-side measures, henceforth indicated by the scenario prefix ‘*Supply_*’. Consequently, seven demand-side measures, and seven combined demand- and supply-side scenarios are compared in this study. The

following supply-side measures are implemented in MESSAGEix-Materials. Note that for scenarios coupled with the full set of supply-side measures, a 2°C climate policy with emission constraints is implemented in MESSAGEix-Materials that includes carbon capture and storage (CCS) measures.

Table S4. Overview table of demand- and supply-side scenarios, underlying CE measures and respective data sources.

Scenario name	Strategy description	Infrastructure-relevant assumptions	Sources
Reference	Reference scenario (no strategies)	Current stocks coupled with SSP2-regional vehicle-km forecasts to project to 2060	MESSAGEix-Transport projection (IIASA, 2024b)
StockConv	Convergence of total infrastructure area to decent infrastructure level	Convergence to ~1,110 m2/cap total infrastructure area by 2060. This threshold corresponds to the level where Human Development Index (HDI) – used a proxy for well-being – is 80% (threshold for high HDI) and HDI begins to saturate (see Figure S7). Shares of individual infrastructure classes in 2060 based on reference scenario. Countries which by 2060 have surpassed the threshold in the reference scenario do not reduce their stocks but instead continue to follow reference scenario trajectories. This approach captures broad empirical patterns without aiming for precise country-level stock-to-wellbeing predictions.	Regression based on HDI data (United Nations Development Programme, 2025)
ShiftToRail	Modal shift from road to rail vehicle-km with a proportional shift in infrastructure	All regions below the average rail share in total vehicle-km (5.5%) of the top six countries with the highest rail share catch up to this level by 2060, with shares of infrastructure classes per mode kept constant	Own assumption
MobReduct	Overall reduction of vehicle-km	Vehicle-km in 2060 reduced in a steplike manner for income regions: 30% reduction for HI, 20% for UMI, 10% for LMI, no reduction for LI. Reduction in road wear extends road lifetimes proportionally, following the same tiered pattern. Such reductions may be realized through via trip avoidance (e.g., through teleworking and locally available services) and reduced trip distances (e.g., through compact urban design).	Own assumption based on Harvey (2013)
CarSharing	Car sharing rate doubled	Parking infrastructure effectively halved by 2060 based on the assumption that car vehicle fleet (two car-owners share one vehicle) and thus demand for capacity is reduced	Own assumption based on Martin et al. (2010)
FleetElect	Private passenger fleet electrification	Expansion of charging and stagnation of conventional fueling stations based on complete shift to electric vehicles by 2060	Own assumption
DemandAll	Combined demand-side measures	Combined measures of scenarios *ShiftToRail, MobReduct, CarSharing* and *FleetElect*	See above
*Supply_**	Combined supply-side measures	Combination with bundle of supply-side measures implemented in MESSAGEix-Materials module: Increased recycling for steel, aluminum, concrete and asphalt, fuel switching, material substitution, and carbon capture and storage (CCS)	Own assumptions implemented in MESSAGEix-Materials (Ünlü et al., 2024)

1.3.1. Demand-side scenarios

Reference scenario

In the reference scenario, material stocks per infrastructure class are coupled with projected vehicle-km (vkm) based on different transportation mode combinations, sources from MESSAGEix-Transport (IIASA, 2024b). For example, base year material stocks for road infrastructure (encompassing various road types, as well as road bridges and tunnels) are projected to 2060 by multiplying the intensity of road material stocks per transport mode (cars and light duty vehicle (LDV), two-wheelers, buses, and trucks) in the base year with the projected vkm. The vkm projections (see Figure S5), smoothened through interpolation and differentiated by regional (Shares socio-economic pathways, 'middle-of-the-road', SSP2) combinations of transport modes (shown in Table S5) were then used as proxies for projecting material stocks into the future.

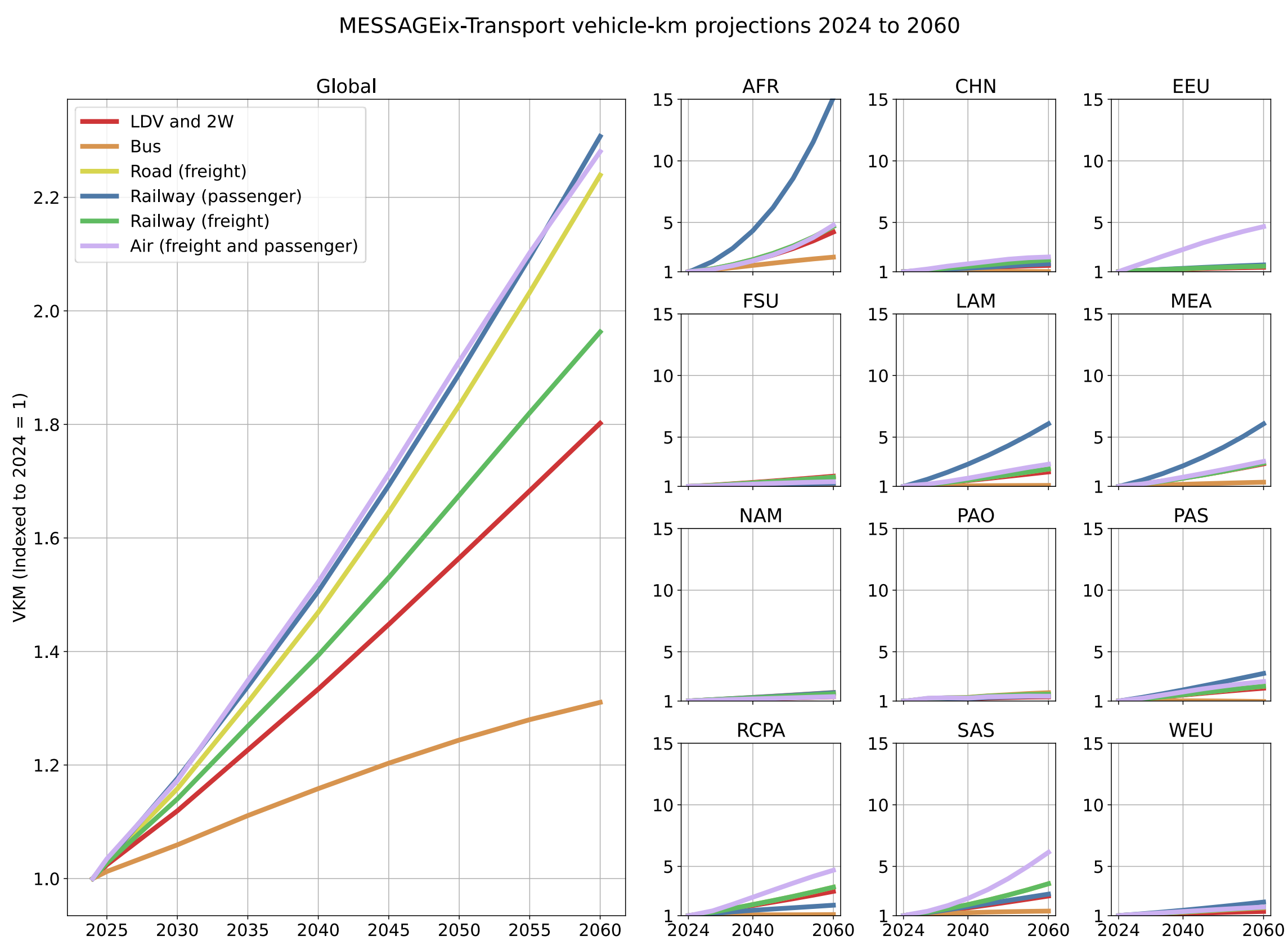

Fig. S5. MESSAGEix-Transport vehicle-km projections from 2024 to 2060 globally and for MESSAGEix regions. SSP2 projections were used as proxies for projecting material stocks to 2060.

Table S5. Overview of infrastructure classes and respective vehicle-km (vkm) data per mode used as proxies for scenario projection.

Infrastructure class	Transport mode proxies
Motorways, primary roads, secondary roads, tertiary roads, local road, rural roads, road bridges and tunnels	Cars and light-duty vehicle (LDV), two-wheeler, bus, truck
Railway, railway bridges and tunnels, railway station, railway platform	Passenger and freight rail
Subway (ground, elevated, underground), tram, other rail, subway station	Passenger rail
Gas station (building and pavement), charging station	Cars and light-duty vehicle (LDV), two-wheeler, bus, truck
Parking (surface, building, underground)	Cars and light-duty vehicle (LDV), two-wheeler
Airport runways	Air transport

Figure S6a shows intensities of total material stocks in mobility infrastructure (roads as major infrastructure type shown exemplarily) per unit of vkm for the MESSAGEix world regions. Intensities vary significantly, implying that the same lengths of roads are withstanding different intensities of use, i.e., more or less traffic depending on the region. Some regions are significantly above average intensity, implying that they have higher degree of available infrastructure for their respective mobility demand, whereas other regions show low intensity with seemingly higher use of available infrastructure. This further implies that regions exhibit different dynamics in regards to how infrastructure evolves with mobility demand, with some regions seemingly expanding their networks in anticipation of demand (high stock/vkm intensity), while others do so rather in reaction to high demand (low intensity).

While these are simplifications of complex real-life dynamics that may depend on a variety of biophysical and socio-economic factors including local environmental conditions, financial constraints, effectiveness of policies, or political stability, they indicate differences in how delayed or anticipated mobility infrastructure networks are expanded in relation to mobility demand. Since the GLOMIS model follows a 'what-if' approach, a linear relationship serving as a proxy for an average relationship was assumed for the reference scenario. Nevertheless, Figure S6b shows back-of-the-envelope re-calculations of the reference scenario if regions above average intensity were to be capped at mean, 25th percentile, or 5th percentile stock-vkm intensities, with the latter roughly corresponding to those of South Asia (SAS) and China (CHN). These caps introduce a deliberate non-linearity into the stock-vkm relationship of above-average regions. They represent futures in which regions with lower utilization of existing infrastructure first increase demand to saturate current capacity before expanding infrastructure stocks.

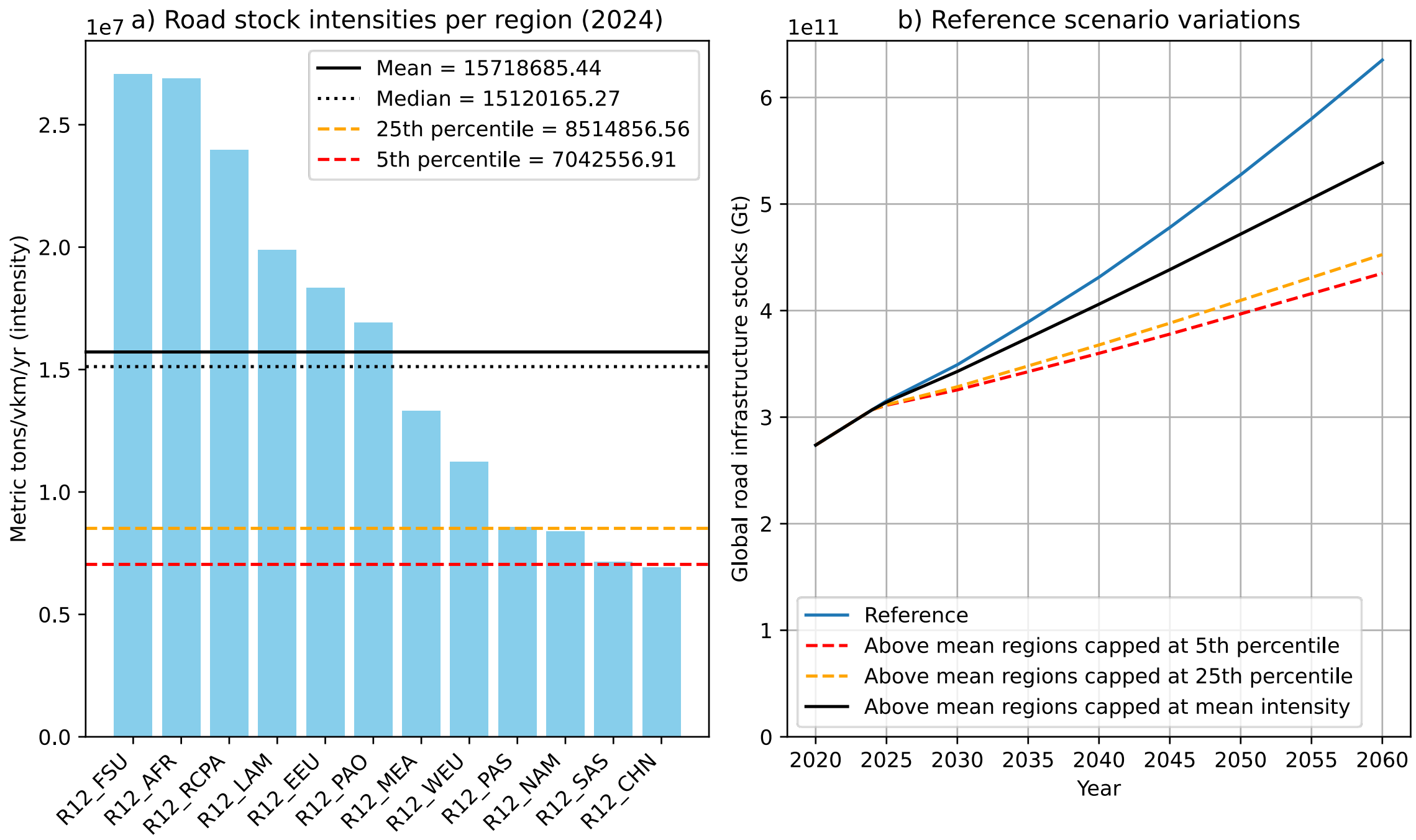

Fig. S6. Material stocks in roads per road-based vkm (LDV, 2W, trucks, buses) intensity in the base year 2024 for the twelve MESSAGEix regions (a) and reference scenario variations for material stocks based on capping of above-mean intensity regions being capped at mean, 25th percentile and 5th percentile intensities (b).

Due to the relatively short timeframe of 35 years (2025-2060), an additional condition set for the reference scenario is that material stocks in mobility infrastructure do not decrease over time. In reality, this is rarely the case and rather than being demolished, infrastructure no longer in use becomes hibernating stock (Hashimoto et al., 2009; Grossegger et al., 2024). For this reason, a model assumption is that only positive stock changes occur as it is highly unlikely that temporary reductions in mobility demand would also lead to reductions in mobility infrastructure within the modelled timeframe.

Decent stock convergence (StockConv)

A *convergence to decent stock level* scenario represents a global convergence to an infrastructure level of 1,112 m^2/cap of total mobility infrastructure area. This corresponds to the average of those national per capita areas falling within a range of ±10% of a human development index (HDI) of 0.8 that the United Nations Development Programme defines as a threshold for very high HDI (United Nations Development Programme, 2025). HDI is herein used as a proxy for decent well-being. The regression method to calculate a decent area (see Figure S7), is based on the methodology used in a predecessor study (Virág et al., 2022). Note that material stocks those countries, which by 2060 have surpassed the threshold in the reference scenario, do not converge negatively, but instead continue to follow reference scenario trajectories.

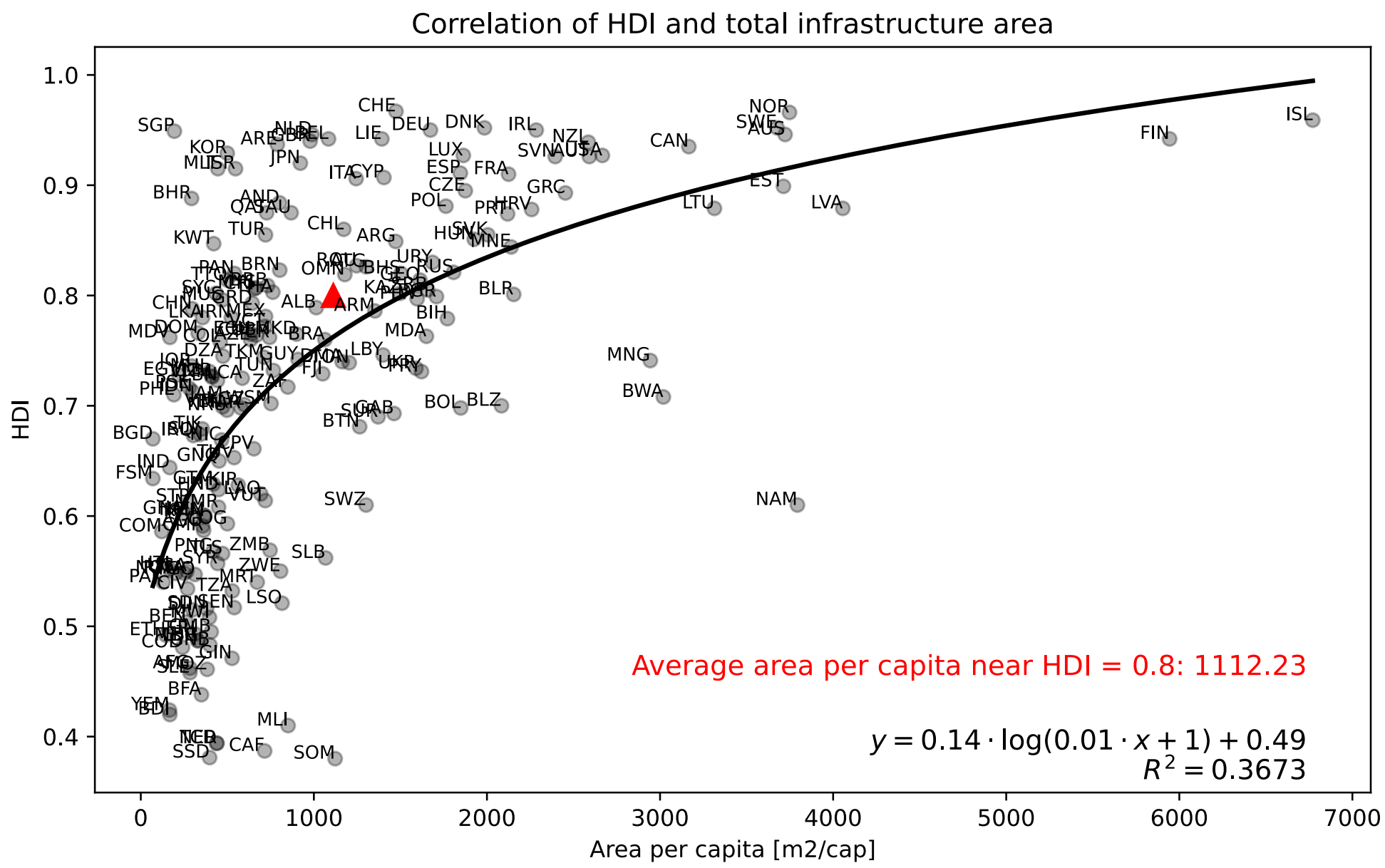

Fig. S7. Correlation of Human Development Index (HDI, 2022 data) and total area per capita (OpenStreetMap, 2024 data). The triangle denotes the average of national-level per capita areas falling within a range of ±10% of an HDI of 0.8.

Using this approach, target per capita stock levels are derived for all countries for 2060. Exponential interpolation is then conducted between 2024 and 2060 per capita stock levels. For this, the Equation 3 is used, where A_t is the interpolated area in a given year, α is the normalized time variable that scales the interpolation across the interval, and *exp* is the exponential function, controlling the rate of change.

$$A_t = A_{2025} + (A_{2060} - A_{2025}) \times \frac{\exp(\alpha) - 1}{\exp(1) - 1} \qquad \text{Equ. 3}$$

Note that in this scenario, shares of infrastructure classes in total per capita area and stocks remain constant over time.

Modal shift to rail (ShiftToRail)

In the modal shift scenario, vkm are shifted from road- to rail-based modes. This is done in three steps. First, similar to the global convergence to decent stock levels scenario, a target value is defined. In this scenario, the share of rail-based vkm in the total vkm per region is used and the average share of the six regions with the highest share of rail-based vkm in total vkm is set as a target to converge vkm to. The regions with the highest share are Centrally Planned Asia excl. China (RCPA: 9.6%), Former Soviet Union (FSU: 8.9%), China (CHN: 5.2%), Pacific OECD (PAO: 3.3%), North America (NAM: 3.2%), and Eastern Europe (2.4%). The average rail share to converge to was calculated as 5.5%.
Second, all countries whose material stocks are below the share in rail in 2060 catch up change to this level. Countries whose rail-share in 2060 is already above this threshold, remain at this level.

Third, current shares of infrastructure types per mode (road, rail, air) are calculated and applied to the modified 2060 shares, thereby keeping the shares of infrastructure types per mode but not overall shares static. Exponential interpolation is then applied between 2024 data and 2060 values, reflecting a gradual increase in the intensity of infrastructure transformation.

Mobility reduction (MobReduct)

In the mobility reduction scenario, total vkm of the reference scenario in the year 2060 are reduced by 30% for high income countries according to World Bank classification region (World Bank, 2024). This is based on an assumption used in Harvey (2013), where a 20% reduction in vkm by 2050 compared to 2007 in the United Kingdom is reported. This tiered approach allows continued mobility and economic development in lower-income regions.
Because vkm likely increased from 2007 to our base year, and because the year of reduction is 2060 and not 2050, raising the mobility reduction rate from 20% to 30% for high income countries was deemed warranted. From high to low-income countries a gradual decrease of reduction rates from 30% to 0% was assumed. Consequently, a 20% reduction was assumed for upper middle-income countries, a 10% for lower middle-income countries and no reduction for low-income countries. Given the correlation between income and mobility levels (Lebrand, 2022), significant reductions in low-income countries could compromise access to essential services. Consequently, mobility of low-income countries was not reduced based on the assumption that these countries require projected mobility levels in order to allow for their economies to grow and catch up to levels found in more affluent countries.
As in the reference scenario, using the infrastructure stocks per vkm intensity, infrastructure stocks for the year 2060 are recalculated. Exponential interpolation is applied to calculate stocks for the years between 2024 and 2060. Note that the model assumes that mobility cannot be lower than base year values. However, this may lead to zero expansion flows, if mobility reduction reaches 2024 levels.
Furthermore, analogous to reduced traffic, lifetimes of roads are increased based on the same rates due to reduced use and wear of roads. For example, if in lower middle-income countries, mobility is assumed to be reduced by 10% by 2060, lifetimes are assumed to be 10% of roads by 2060.

Increased car sharing (CarSharing)

In the increased car sharing scenario, it is assumed that two people will share one car by the year 2060, meaning that the vehicle fleet will effectively be halved (Martin et al., 2010). Consequently, it is implied that parking space requirements will likewise effectively be halved by this year. This affects surface parking as well as parking buildings and underground parking structures.

Vehicle fleet electrification (FleetElect)

In the vehicle fleet electrification scenario, it is assumed that the entire vehicle fleet is completely electrified by the year 2060. The scenario therefore assumed that by

2060, no more gas stations will be built. Instead, charging stations are solely responsible for fueling the vehicle fleet, requiring a continuous expansion of e-charging infrastructure. New construction of conventional gas stations is slowly being phased out.

Combined demand-side measures (DemandAll)

The *DemandAll* scenario combines the preceding four demand-side scenario measures but excludes StockConv where specific stock levels are targeted and a combination is not viable. Note that not all demand-side measures yield net reductions in total material stocks. In the case of the modal shift to rail, total stocks increase due to the expansion of the railway network. This is counterbalanced by other measures such as a reduction in overall mobility (MobReduct).

1.3.2. Supply-side

Material substitution

The material substitution supply-side scenario allows for substitution of cement clinker and bitumen with innovative materials. Clinker can be substituted with calcined clays and limestone. The substitution ratio is set according to the results of Cancio Díaz et al. (2017). In bitumen production, a bio-based production route is available using lignin by-product from paper production (Gaudenzi et al., 2023). The regional availability of lignin is projected by estimating future paper production.

Increased recycling

The recycling scenario allows increasing recycling rates for the commodities considered in this study. Based on recently reported values, the starting values for recycling rates are 85% for steel and 90% for aluminum. In concrete mixing, 30% of primary aggregates can be replaced via secondary concrete. Asphalt is a mixture of 5% bitumen and 95% aggregates (sand and gravel). In default recycling, 25% of primary aggregates in asphalt can be replaced by secondary, downcycled asphalt while bitumen is not replaced.
With increased recycling assumptions, recycling rates of both steel and aluminum are increased to 98%. Recycling of concrete is changed to allow for a 60% replacement of primary aggregates with secondary, downcycled concrete. For asphalt, up to 50% of aggregates can be recycled from EOL asphalt with a reduction in bitumen due to the addition of chemicals. For the parameterization of current asphalt mix and recycling options, Bruno et al. (2024) was used.

Fuel switching

For cement, iron and steel, and aluminum industries the high and low temperature heat need can be satisfied via different fuels. These include fossil fuels such as coal, oil, gas, as well as renewables such as biomass, hydrogen, solar, electricity, and methanol.
The fuel switching scenario allows the model to choose, which fuels to use in heavy industry. MESSAGEix-Materials represents heat supply with either generalized

furnace and boiler technologies or process-specific technologies. This includes hydrogen use for direct-reduction of iron and cement kilns, as well as biomass use and electrification of high-temperature heat supply for cement and aluminum production. All other scenarios are constrained to maintain current regional fuel use patterns.

Carbon capture and storage

Carbon capture and storage (CCS) technologies are included for cement and iron and steel industries. The CCS scenario allows the model to retrofit cement clinker kilns, blast furnaces and iron direct-reduction plants with carbon capture technology. In the iron and steel industry, the blast furnaces can be equipped with CCS combined with top gas recirculation which enables more efficient gas capture and fuel use. In the cement industry, kilns are equipped with CCS technologies which can both capture process- and combustion-related emissions.

1.4. Data sources

OpenStreetMap (downloaded via Geofabrik, 2024) is used as inventory data for all infrastructure classes but airport runways, for which an additional, more comprehensive crowd-sourced dataset was identified (OurAirports, 2024). The extent of the global road network was estimated to be more than 80% complete in the year 2017 (Barrington-Leigh & Millard-Ball, 2017) and can be expected to have further improved in coverage since.

A dataset of regional MI factors and widths compiled for the predecessor study (Wiedenhofer, Baumgart, et al., 2024) is expanded by including data for France, Denmark, and Sweden. For countries, for which no specific inputs were compiled, global averages are used. For lifetimes, global factors from literature are used.

Mode-specific mobility projections from MESSAGEix-Transport (IIASA, 2024b) were used as proxies for projecting mobility stocks in the reference scenario. Because data outputs from MESSAGEix are available for every five years, annual data was linearly interpolated. Likewise, for population, SSP2 projections from MESSAGEix were used (IIASA, 2024a).

Allocation of countries to income levels, i.e., high-, upper middle-, lower middle-, and low-income was done based on 2023 World Bank data (World Bank, 2024).

1.5. Uncertainty

Uncertainty is assessed using ranges for MI factors, widths, and lifetimes. For MI factors and widths, the interquartile range of data points (25th to 75th percentiles) is used as lower and upper bound inputs. Lifetimes are varied by ±20% for low and high estimate inputs. Averages across all data points serve as best-guess inputs. Because some data points for MI factors and widths show very high fluctuation for individual infrastructure classes, a divergence of ±30% from the mean is used as a limitation to lower and upper bound inputs.

1.6. Bitumen consumption

Comparing material consumption modelled in GLOMIS with consumption reported in statistics is challenging. If we compare GLOMIS annual bitumen consumption (based on a 5% bitumen content in asphalt assumption) with bitumen consumption data reported by the United Nations Statistics Division (2024) (see Figure S8), we find most disagreement in Sub-Saharan Africa (AFR, orange) and Latin-America and Caribbean (LAM, purple) countries. This may be due to incomplete reporting in statistics, or an overestimation of road quality and asphalt consumption in these regions, as data for material intensity factors are mainly from higher-income countries (see section 1.7).

On the contrary, for countries with higher material flows such as USA, China, and Russia, modelled and reported data seem to match significantly better. Note that bitumen consumption refers to primary material consumption and does not consider secondary bitumen flows. Consequently, for the purpose of comparison, we assumed secondary material shares per income region identical to those used in Wiedenhofer et al. (2024).

Besides differences in completeness (both in terms of OpenStreetMap and at the statistical level), reasons for differences in bitumen consumption estimates can be non-representative material intensity factors and lifetime assumptions. Regardless of the individual reasons for divergence in data, bottom-up material stock and flow modelling as conducted herein is known to yield results that can differ significantly from those modelled using inflow-driven methods, as well as from statistical data (Streeck et al., 2024)

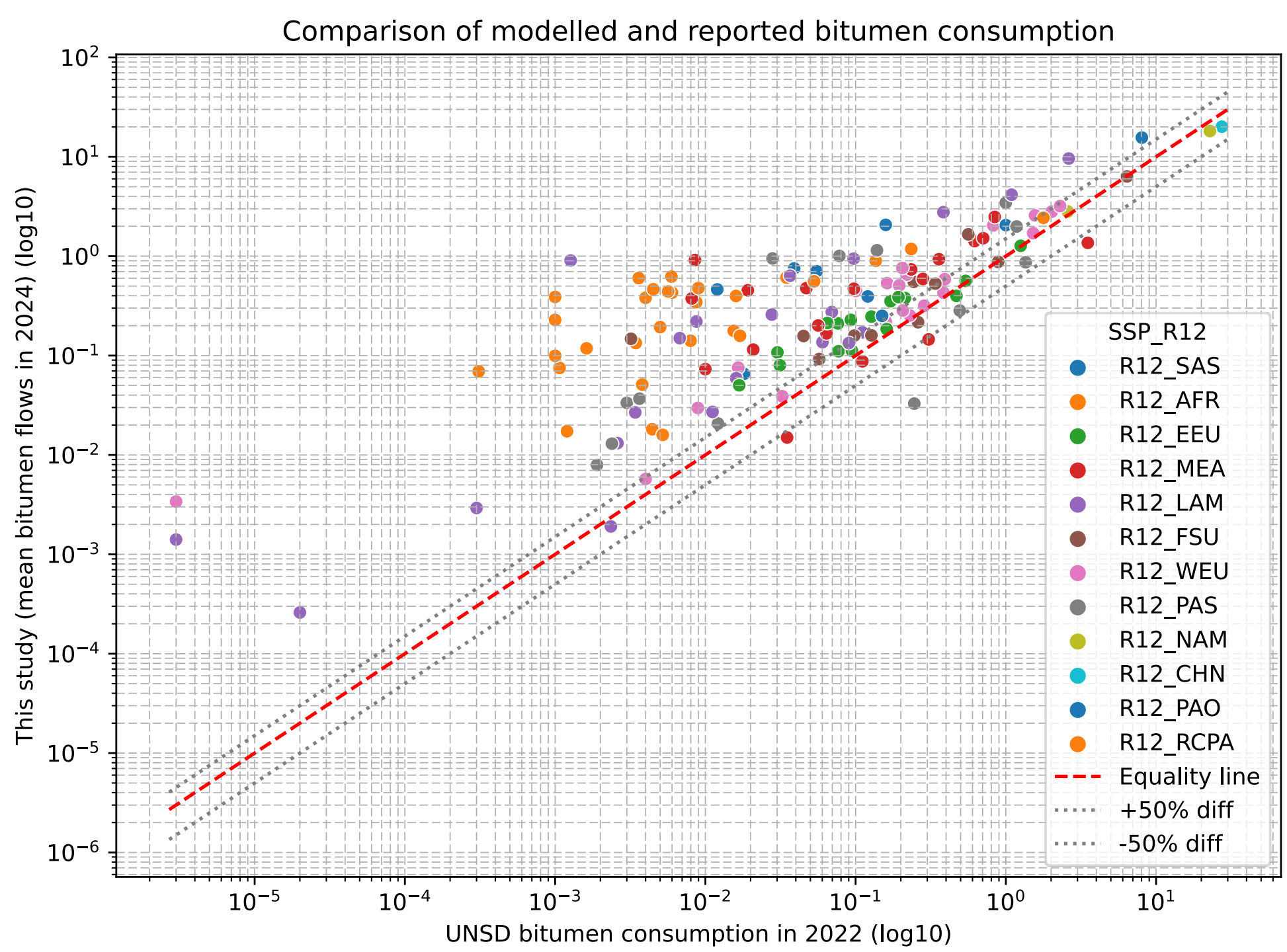

Fig. S8. Scatterplot comparison of national annual bitumen consumption: Modelled bitumen flows (expansion, maintenance and replacement) in 2024 vs. bitumen consumption in 2022 as reported by the United Nations Statistics Division (2024).

1.7. Study limitations

Some limitations related to model data inputs and scenario design remain. For one, the current scenario design is based on national-level data, instead of a spatially explicit analysis of infrastructure patterns. Due to the lack of spatial explicitness, there is little information on subnational distribution of material stocks in mobility infrastructure that could otherwise be used in the scenario design. This impedes a spatial analysis of subnational stock dynamics, which is beyond the scope of this paper.

Second, there is some bias towards higher-income regions in data inputs, as the compiled MI factors and widths are mostly available for countries of the Global North. The main criterion for selecting data was a hierarchical classification analogous to infrastructure class hierarchy (e.g., different road classes). Likewise, OSM data was shown to be more complete in higher-income countries, and especially in urban areas (Minaei, 2020; Herfort et al., 2023).

Third, the use of vehicle-km for projecting material stocks assumes a direct link between material stock stocks and mobility increases. However, the actual relationship is not yet understood well enough to derive a relational function. Nevertheless, the vkm-coupled stock projection, though a simplified approach, are deemed sufficient for a first estimate of future stocks.

2. Additional model results

2.1. Material stock projections

Material stocks results as shown in manuscript Figure 3a are disaggregated by main infrastructure classes in Figures S9 and S10. Representing all car-related infrastructure, road-based infrastructure (Figure S9) encompasses all roads, road bridges and tunnels, as well as parking, fueling and charging infrastructure. Compared to the reference scenario, the StockConv scenario almost doubles material stocks in global road infrastructure by 2060.

Rail-based infrastructure (Figure S10) encompasses all railway lines, railway bridges and tunnels, subways, trams, as well as railway and subway station buildings and platforms. In contrast to road-infrastructure, the main driver of stock increases is ShiftToRail, both as a standalone measure, and as part of the DemandAll demand-side measure bundle.

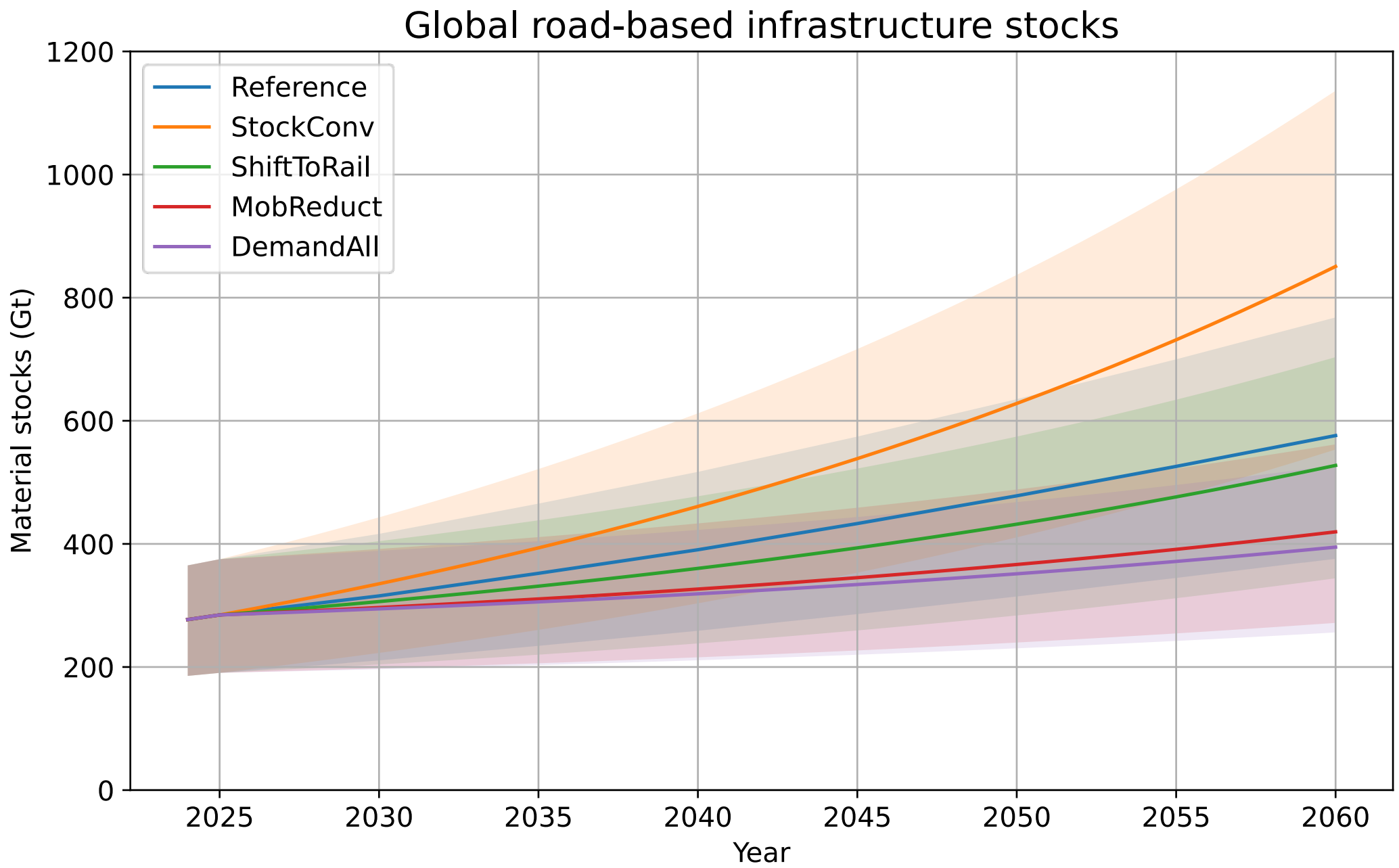

Fig. S9. Road-based infrastructure material stock projections for the reference and demand-side scenarios. Road-based infrastructure encompasses all types of car-related infrastructure, includes various road types, road bridges and tunnels, parking, fueling/charging infrastructure. Uncertainty ranges reflect 25th and 75th percentiles of data points for widths and material intensity factors.

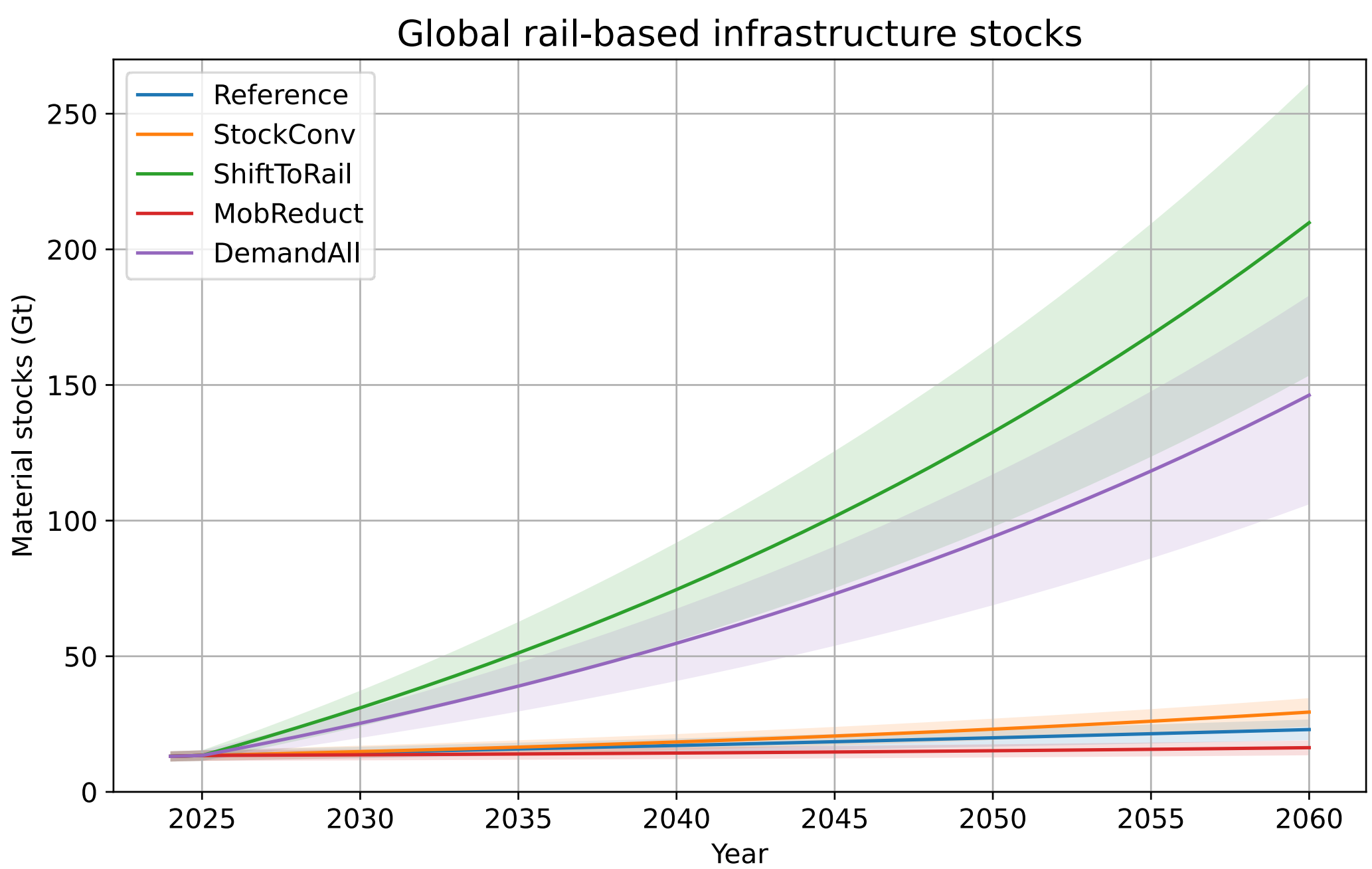

Fig. S10. Rail-based infrastructure material stock projections for the reference and demand-side scenarios. Rail-based infrastructure includes railway, railway bridges and tunnels, subways, trams, other railway, as well as railway and subway station buildings and platforms. Uncertainty ranges reflect 25th and 75th percentiles of data points for widths and material intensity factors.

Note that in both Figure S9 and S10, scenarios CarSharing and FleetElect are not shown as they do not differ significantly from the reference scenario. This is because relevant infrastructure categories (i.e., parking, fueling, and charging infrastructure) show stock levels considerably lower than those of the remaining stock types (i.e., roads, Figure S9), or because they are simply not relevant for the main infrastructure class (not relevant for rail, Figure S10).
As shown in manuscript Figure 3b, material stock scenario trajectories vary across world regions. Similarly, Figure S11 shows material stock developments for different scenarios vary across income levels. Results how that for high-income countries, a modal shift to rail (ShiftToRail) drives material stocks more than stock convergence (StockConv) does, with the latter yielding stock levels similar to the reference scenario. For upper middle-income countries, StockConv surpasses ShiftToRail as the primary driver of material stocks, although both scenarios have a significantly higher stock increase by 2060 compared to high-income countries. For lower middle- and low-income countries, the stock expansion in StockConv is considerably more material-intensive than other scenarios, reflecting that the catch-up to ‘decent’ stock levels occurs primarily in the economically developing regions.

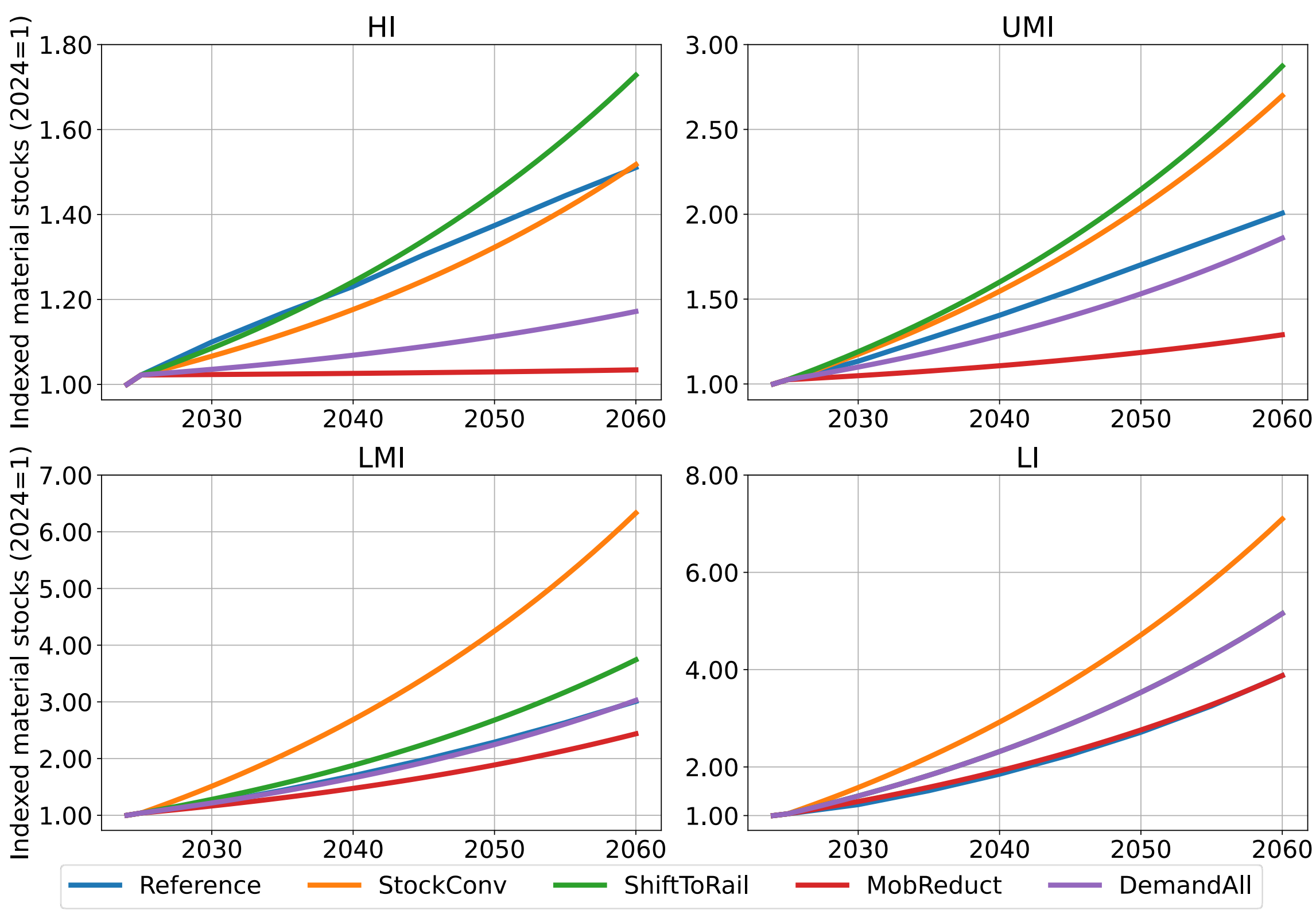

Fig. S11. Indexed mean-estimate total mobility infrastructure material stock projections for the four World Bank income levels (World Bank, 2024). Scenarios CarSharing and FleetElect are not visibly distinguishable from the reference scenario and consequently not shown. Abbreviations: HI = High-income, UMI = Upper middle-income, LMI = Lower middle-income, LI = Low-income.

2.2. Annual material flows

Global total annual material flows associated with mobility infrastructure expansion, maintenance and replacement are shown in Figure S12. These flows are demand inputs to MESSAGEix-Materials where embodied emissions are calculated (see Figure S1).

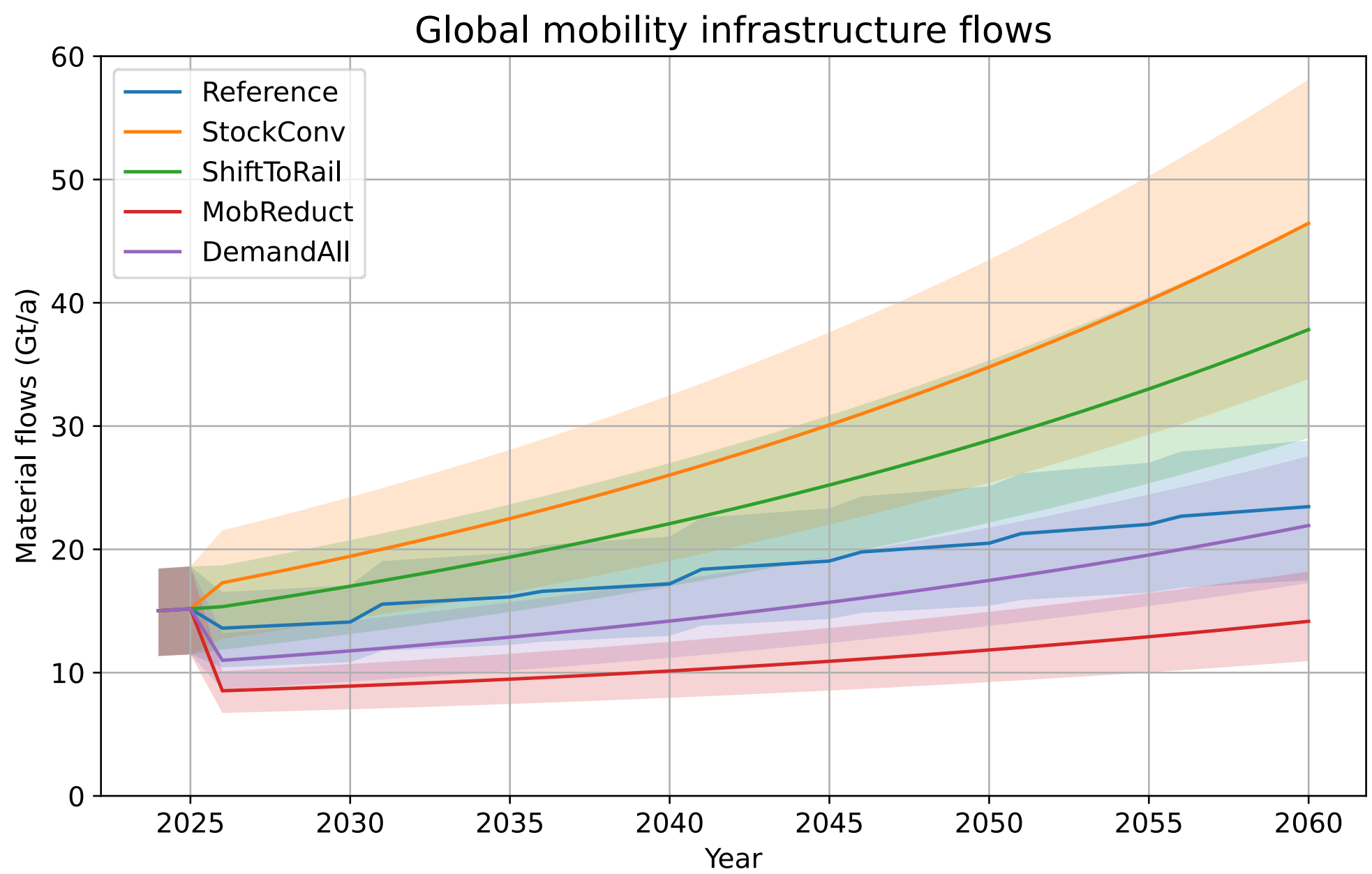

Fig. S12. Total material flows (expansion, maintenance, and replacement). Note that for material stocks, scenarios CarSharing and FleetElect are not visibly distinguishable from the reference scenario and consequently not shown. Uncertainty ranges reflect 25th and 75th percentiles of data points for widths and material intensity factors.

Figure S13 shows total material flows due to expansion of global mobility infrastructure. Figure S14 shows total material flows due to maintenance and EOL replacement of global mobility infrastructure stocks.

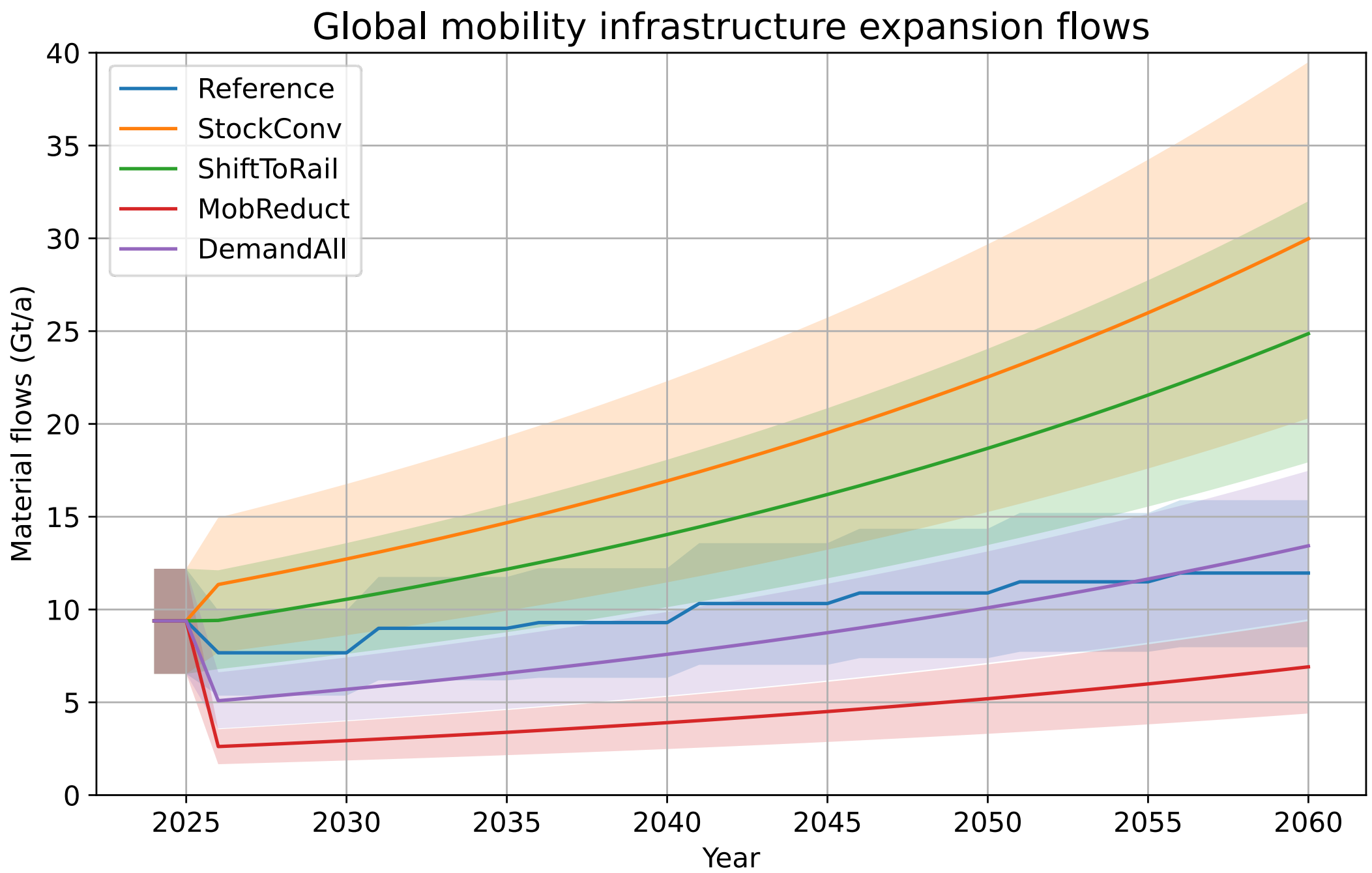

Fig. S13. Total material flows associated with expansion of mobility infrastructure. Uncertainty ranges reflect 25th and 75th percentiles of data points for widths and material intensity factors.

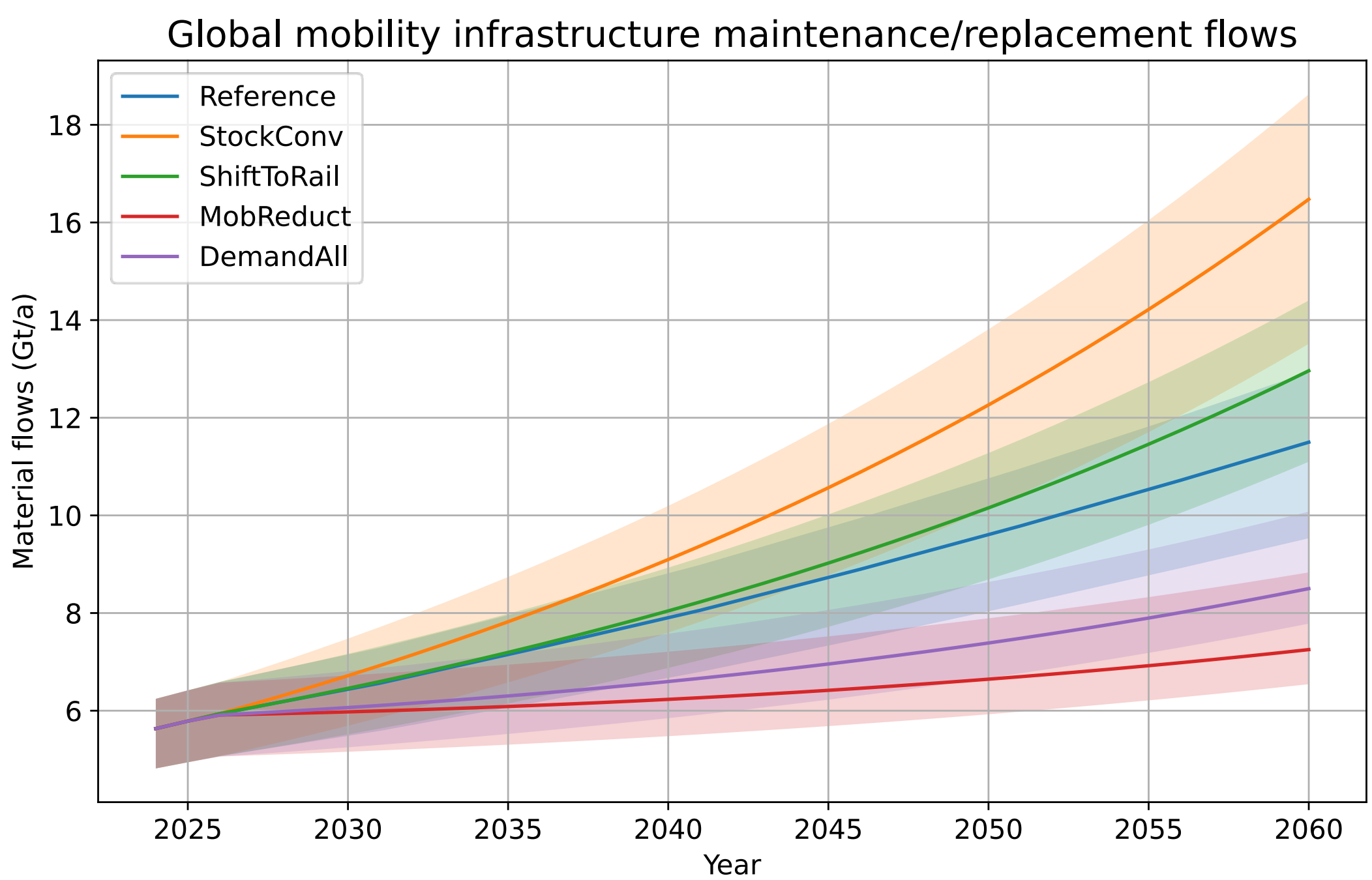

Fig. S14. Total material flows associated with maintenance and replacement of mobility infrastructure. Uncertainty ranges reflect 25th and 75th percentiles of data points for widths and material intensity factors.

Figure S15 shows total material flows due to maintenance and replacement of global mobility infrastructure. Compared to expansion, maintenance and replacement is responsible for roughly 50% fewer annual mass flows, albeit with between 6 and 20 Gt/yr, maintenance and replacement flows are substantial. Trends across scenarios are similar for both flow types.

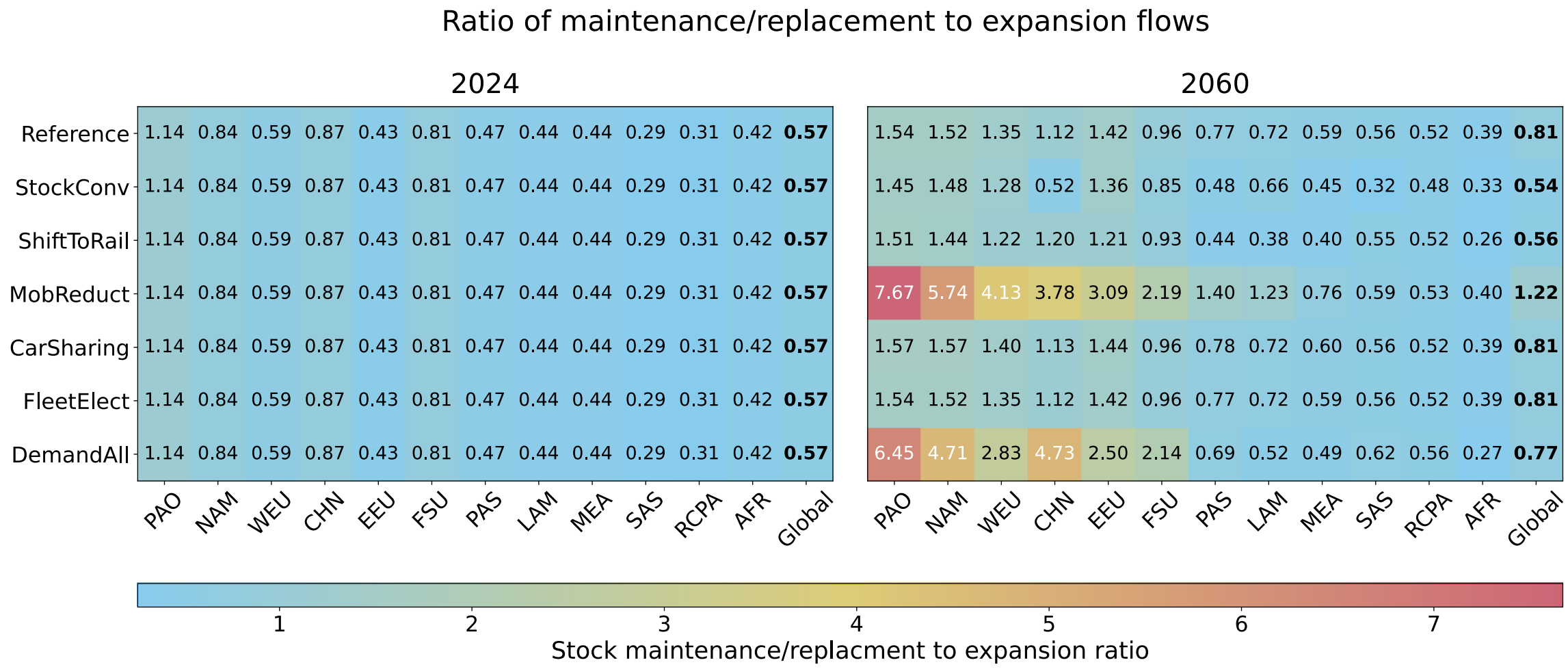

Fig. S15. Ratio of maintenance and replacement flows per scenario and region for the years 2025 and 2060. The higher the value, the higher the share of maintenance/replacement in total flows.

2.3. Material stock distribution across the globe

The ratio of maintenance and replacement to expansion flows changes over time with variation across scenarios and regions, as can be seen in Figure S15, where lower values correspond to a lower share of infrastructure expansion in total flows and a value of 1 indicating equal contribution to material flows by expansion and maintenance/replacement activities. Particularly in scenarios (and regions) where expansion is curbed by mobility reductions and stocks begin to saturate, maintenance and replacement of mobility infrastructure stocks become considerably more relevant, reaching ratios of almost 8.

Looking at country-level changes in material stocks from 2024 to 2060, we see that in the reference scenario (Figure S16a), most change occurs in Eastern Europe, Southern Africa, where an increase to 150 t/cap can be observed due to mobility increases. In the StockConv scenario (Figure S16b), with a global convergence of per capita mobility infrastructure recently developed or developing and/or high-population density regions such China, South and Southeast Asia, and Central Africa show considerably higher per capita stock additions.

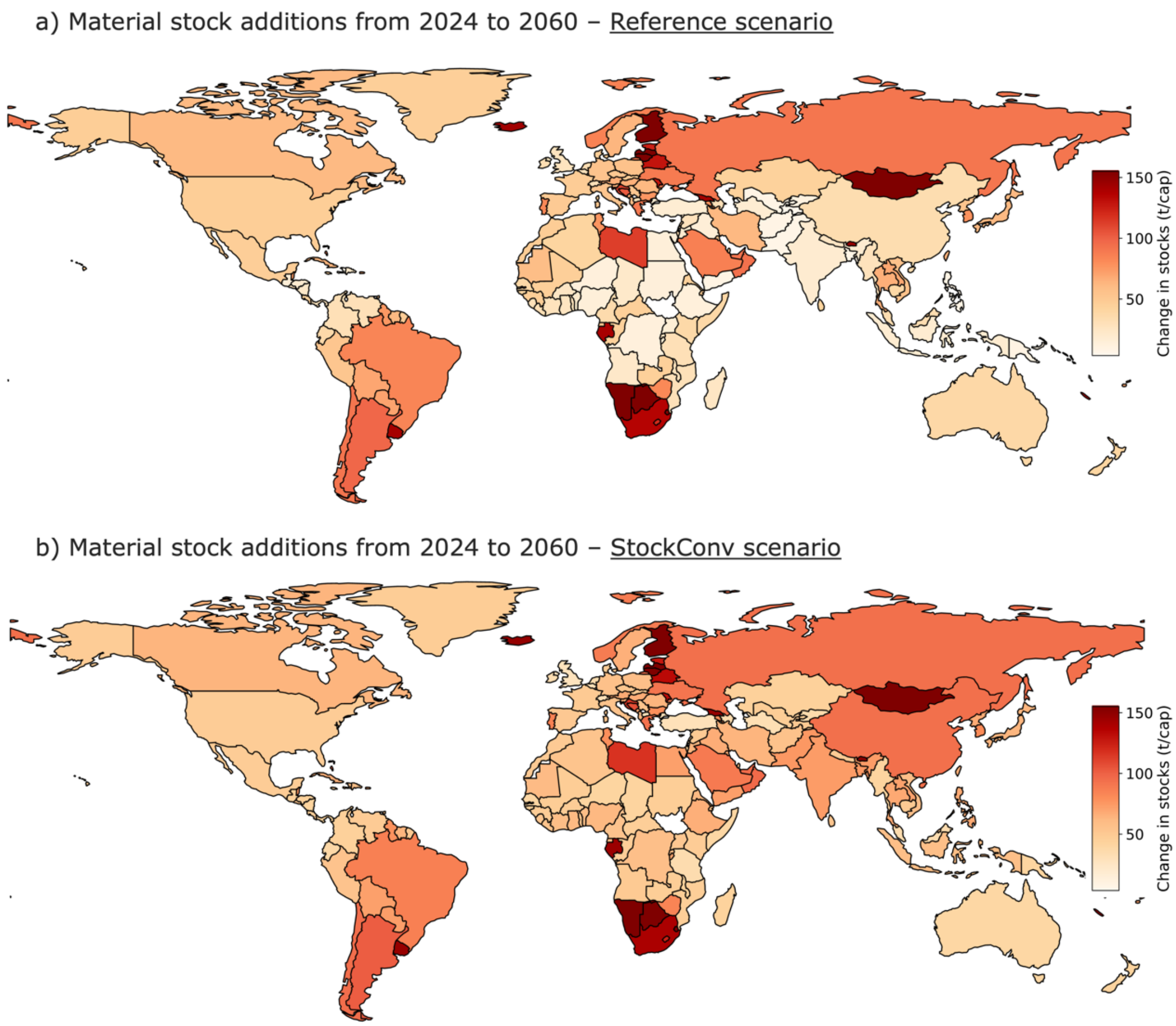

Fig. S16. Material additions to per capita stock sin mobility infrastructure from 2024 to 2060 in the reference (a) and StockConv scenario (b).

Gini coefficients for material stock distribution are shown in Figure S17. By 2060, the Gini remains at 2024 levels in both in the reference and the ShiftToRail scenarios. In the StockConv scenario, however, Gini coefficients would be lowered considerably from 0.48 in 2024 to 0.22 in 2060 in the StockConv scenario. Remaining inequality can be attributed to some countries already having surpassed the decent per-capita stock threshold.

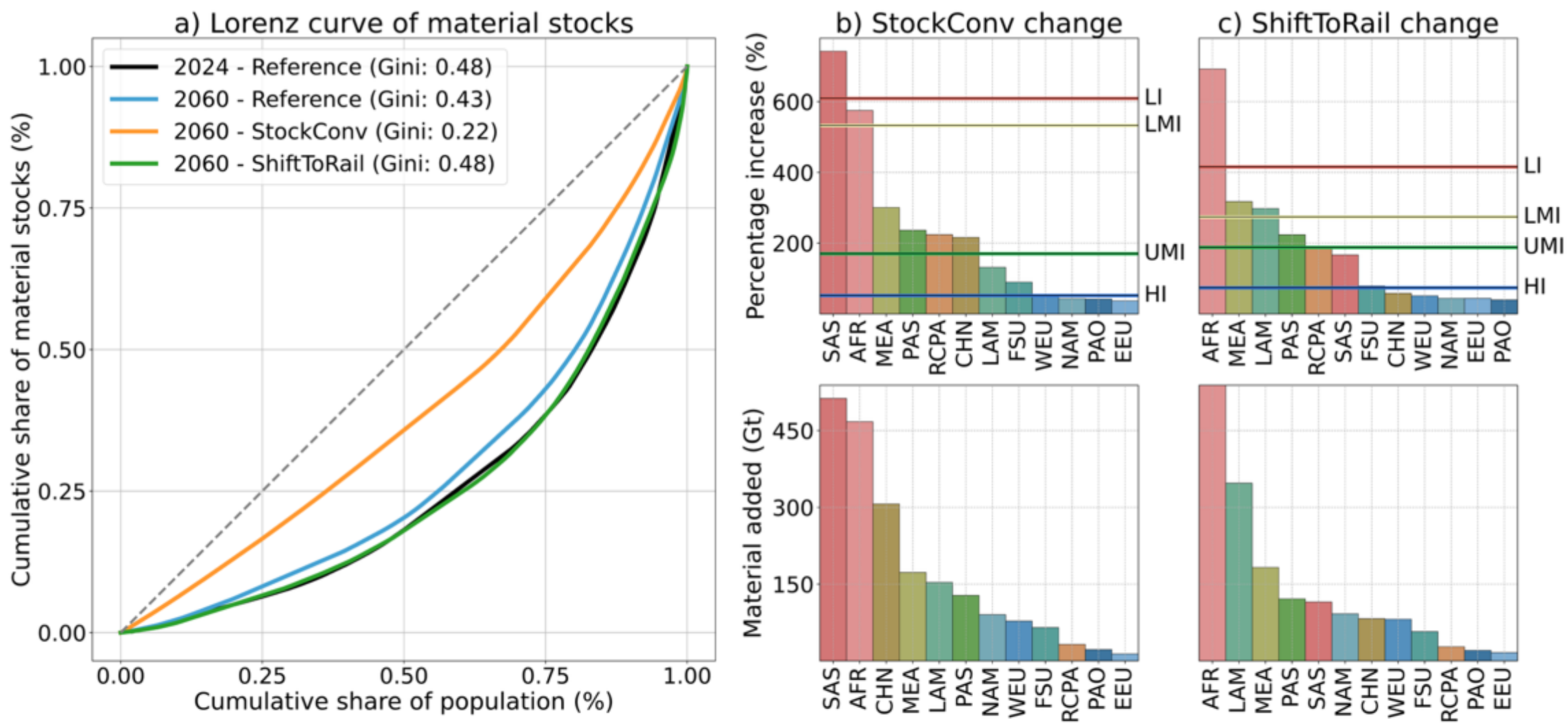

Fig. S17. Material additions to per capita stock sin mobility infrastructure from 2024 to 2060 in the reference (a) and StockConv scenario (b).

In the StockConv scenario, South Asia (SAS) and Sub-Saharan Africa (AFR) see the highest percentage increase in stocks, with an 8- and 7-fold increase by 2060, respectively (Figure S17b). Bangladesh, Pakistan, Ethiopia, Nigeria, and countries in the East-African Great Lakes region are the countries with the highest percentage change. AFR and SAS dominate stock additions in absolute terms, followed by China. The countries with the highest absolute stock increases are India with 350 Gt of material added to stocks, followed by China (+307 Gt), Nigeria (+94 Gt), and Pakistan (+94 Gt).
In the ShiftToRail scenario, Latin America (LAM) and Middle East & North Africa (MEA) are main drivers of stock growth, albeit with half of the 7-fold growth in AFR (Figure S17c). In regard to absolute stock increases, South Africa leads with 151 Gt of stocks added, followed by Brazil, India, and China.

2.4. Global carbon budget

We compare the modelled embodied GHG emissions of the mobility infrastructure with the global carbon budget remaining to keep global warming below 1.5 and 2°C, which was 250 and 1,200 $GtCO_2$ in 2023, respectively (Lamboll et al., 2023). Considering the global CO_2 emissions (IEA, 2025) between the time of this estimate (January 2023) and the start of our scenarios (January 2025), the updated remaining global carbon budget is 175 and 1,125 $GtCO_2$ for the 1.5 and 2°C targets, respectively. Our mean-estimate reference scenario without supply-side mitigation measures would use up 1.0 to 6.2% (for a 2°C and 1.5°C, respectively) of the global carbon budget. A StockConv scenario could require 1.7% (2°C) or 10.9% (1.5°C) of the remaining global carbon budget. In the ShiftToRail scenario, 3.3% or 21.2% are required, primarily driven by increased concrete and steel consumption, both used extensively in rail-based infrastructure. In MobReduct, 0.5 or 3.5% would be locked in. With modal shift and vehicle-km reduction balancing each other out in terms of embodied emissions, DemandAll

locks in roughly as much as the reference scenario. The reference scenario with only supply-side measures (Supply_Reference) would lock in 0.6 or 3.8%. This share is 2.1 or 13.3% and 0.3 or 2.2% in Supply_ShiftToRail and Supply_MobReduct, respectively, while combining all supply- and demand-side measures (Supply_DemandAll) would lock in 1.3 or 8.6%.

3. References

Alzaim, M., Gedik, A., & Lav, A. H. (2020). Effect of Modulus of Bituminous Layers and Utilization of Capping Layer on Weak Pavement Subgrades. *Civil Engineering Journal*, *6*(7), 1286–1299. https://doi.org/10.28991/cej-2020-03091548

Alzard, M. H., Maraqa, M. A., Chowdhury, R., Khan, Q., Albuquerque, F. D. B., Mauga, T. I., & Aljunadi, K. N. (2019). Estimation of Greenhouse Gas Emissions Produced by Road Projects in Abu Dhabi, United Arab Emirates. *Sustainability*, *11*(8). https://doi.org/10.3390/su11082367

Antoniou, F., Aretoulis, G., Giannoulakis, D., & Konstantinidis, D. (2023). Cost and Material Quantities Prediction Models for the Construction of Underground Metro Stations. *Buildings*, *13*(2), 382. https://doi.org/10.3390/buildings13020382

Augiseau, V., & Kim, E. (2021). Spatial characterization of construction material stocks: The case of the Paris region. *Resources, Conservation and Recycling*, *170*, 105512. https://doi.org/10.1016/j.resconrec.2021.105512

Bai, J., Qu, J., Maraseni, T. N., Wu, J., Xu, L., & Fan, Y. (2019). Spatial and Temporal Variations of Embodied Carbon Emissions in China's Infrastructure. *Sustainability*, *11*(3). https://doi.org/10.3390/su11030749

Barrington-Leigh, C., & Millard-Ball, A. (2017). The world's user-generated road map is more than 80% complete. *PLOS ONE*, *12*(8), e0180698. https://doi.org/10.1371/journal.pone.0180698

Bruno, S., Loprencipe, G., Di Mascio, P., Cantisani, G., Fiore, N., Polidori, C., Riccio, G., D'Andrea, A., & Moretti, L. (2024). Eco-efficient asphalt recycling for urban slow mobility. *Euro-Mediterranean Journal for Environmental Integration*, *9*(2), 957–964. https://doi.org/10.1007/s41207-024-00524-0

Cancio Díaz, Y., Sánchez Berriel, S., Heierli, U., Favier, A. R., Sánchez Machado, I. R., Scrivener, K. L., Martirena Hernández, J. F., & Habert, G. (2017). Limestone calcined clay cement as a low-carbon solution to meet expanding cement demand in emerging economies. *Development Engineering*, *2*, 82–91. https://doi.org/10.1016/j.deveng.2017.06.001

Chen, J., Zhao, F., Liu, Z., Ou, X., & Hao, H. (2017). Greenhouse gas emissions from road construction in China: A province-level analysis. *Journal of Cleaner Production*, *168*, 1039–1047. https://doi.org/10.1016/j.jclepro.2017.08.243

Cruz, M. (2016). *Material stock of infrastructure. Comparative analysis between Swedish and Mexican cities* [Chalmers University of Technology]. https://odr.chalmers.se/server/api/core/bitstreams/f22cff06-0817-4ea4-a342-094a490de1ba/content

CSIR. (2000). *Guidelines for human settlement planning and design: The red book*. Council for Scientific and Industrial Research; ResearchSpace. http://hdl.handle.net/10204/3750

DMR. (2014). *Pavement Design Guidelines (Flexible Pavement)*. Department of Roads, Ministry of Physical Infrastructure and Transport, Government of Nepal. https://dor.gov.np/home/publication/general-documents/dor-pavement-design-guidelines

DMT. (2016). *Pavement Design Manual* (TR-513). Department of Municipal Affairs and Transport, Government of Abu Dhabi. https://jawdah.qcc.abudhabi.ae/en/Registration/QCCServices/Services/STD/ISGL/ISGL-LIST/TR-513.pdf

Eliassen, A. R., Faanes, S., & Bohne, R. A. (2019). Comparative LCA of a concrete and steel apartment building and a cross laminated timber apartment building. *IOP Conference Series: Earth and Environmental Science*, *323*(1), 012017. https://doi.org/10.1088/1755-1315/323/1/012017

Frantz, D., Schug, F., Wiedenhofer, D., Baumgart, A., Virág, D., Cooper, S., Gómez-Medina, C., Lehmann, F., Udelhoven, T., van der Linden, S., Hostert, P., & Haberl, H. (2023). Unveiling patterns in human dominated landscapes through mapping the mass of US built structures. *Nature Communications*, *14*(1), Article 1. https://doi.org/10.1038/s41467-023-43755-5

Gaudenzi, E., Cardone, F., Lu, X., & Canestrari, F. (2023). The use of lignin for sustainable asphalt pavements: A literature review. *Construction and Building Materials*, *362*, 129773. https://doi.org/10.1016/j.conbuildmat.2022.129773

Geofabrik. (2024). *OpenStreetMap Data Extracts*. https://download.geofabrik.de/

Grossegger, D., MacAskill, K., & Al-Tabbaa, A. (2024). A critical review of road network material stocks and flows: Current progress and what we can learn from it. *Resources, Conservation and Recycling*, *205*, 107584. https://doi.org/10.1016/j.resconrec.2024.107584

Haberl, H., Baumgart, A., Zeidler, J., Schug, F., Frantz, D., Palacios-Lopez, D., Fishman, T., Peled, Y., Cai, B., Virág, D., Hostert, P., Wiedenhofer, D., & Esch, T. (2024). *Weighing the Global Built Environment: High Resolution Mapping and Quantification of Material Stocks in Buildings* (SSRN Scholarly Paper 4879630). Social Science Research Network. https://doi.org/10.2139/ssrn.4879630

Haberl, H., Wiedenhofer, D., Schug, F., Frantz, D., Virág, D., Plutzar, C., Gruhler, K., Lederer, J., Schiller, G., Fishman, T., Lanau, M., Gattringer, A., Kemper, T., Liu, G., Tanikawa, H., van der Linden, S., & Hostert, P. (2021). High-Resolution Maps of Material Stocks in Buildings and Infrastructures in Austria and Germany. *Environmental Science & Technology*, *55*(5), 3368–3379. https://doi.org/10.1021/acs.est.0c05642

Harvey, L. D. D. (2013). Global climate-oriented transportation scenarios. *Energy Policy*, *54*, 87–103. https://doi.org/10.1016/j.enpol.2012.10.053

Hashimoto, S., Tanikawa, H., & Moriguchi, Y. (2009). Framework for estimating potential wastes and secondary resources accumulated within an economy – A case study of construction minerals in Japan. *Waste Management*, *29*(11), 2859–2866. https://doi.org/10.1016/j.wasman.2009.06.011

Henderson, M., & van Zyl, G. (2017, July 10). *Management of unpaved roads: Developing a strategy and refining models*. 36th Annual Southern African Transport Conference, Pretoria, South Africa. http://hdl.handle.net/2263/62740

Herfort, B., Lautenbach, S., Porto de Albuquerque, J., Anderson, J., & Zipf, A. (2023). A spatio-temporal analysis investigating completeness and inequalities of global urban building data in OpenStreetMap. *Nature Communications*, *14*(1), Article 1. https://doi.org/10.1038/s41467-023-39698-6

IEA. (2025). *Global Energy Review 2025: CO2 Emissions*. https://www.iea.org/reports/global-energy-review-2025/co2-emissions

IIASA. (2024a). *MESSAGEix Population and GDP*. https://docs.messageix.org/projects/global/en/stable/socio_econ/pop_GDP.html

IIASA. (2024b). *MESSAGEix-Transport*. https://docs.messageix.org/projects/models/en/stable/transport/index.html

JICA. (2018). *Guideline for Design of Road Tunnel*. Japan International Cooperation Agency. https://openjicareport.jica.go.jp/pdf/12303566.pdf

Lamboll, R. D., Nicholls, Z. R. J., Smith, C. J., Kikstra, J. S., Byers, E., & Rogelj, J. (2023). Assessing the size and uncertainty of remaining carbon budgets. *Nature Climate Change*, *13*(12), 1360–1367. https://doi.org/10.1038/s41558-023-01848-5

Lanau, M., & Liu, G. (2020). Developing an Urban Resource Cadaster for Circular Economy: A Case of Odense, Denmark. *Environmental Science & Technology*, *54*(7), 4675–4685. https://doi.org/10.1021/acs.est.9b07749

Lucas, A., Alexandra Silva, C., & Costa Neto, R. (2012). Life cycle analysis of energy supply infrastructure for conventional and electric vehicles. *Energy Policy*, *41*, 537–547. https://doi.org/10.1016/j.enpol.2011.11.015

Martin, E., Shaheen, S. A., & Lidicker, J. (2010). Impact of Carsharing on Household Vehicle Holdings: Results from North American Shared-Use Vehicle Survey. *Transportation Research Record*, *2143*(1), 150–158. https://doi.org/10.3141/2143-19

Miatto, A., Dawson, D., Nguyen, P. D., Kanaoka, K. S., & Tanikawa, H. (2021). The urbanisation-environment conflict: Insights from material stock and productivity of transport infrastructure in Hanoi, Vietnam. *Journal of Environmental Management*, *294*, 113007. https://doi.org/10.1016/j.jenvman.2021.113007

Minaei, M. (2020). Evolution, density and completeness of OpenStreetMap road networks in developing countries: The case of Iran. *Applied Geography*, *119*, 102246. https://doi.org/10.1016/j.apgeog.2020.102246

Mulrow, J., & Grubert, E. (2023). Greenhouse gas emissions embodied in electric vehicle charging infrastructure: A method and case study of Georgia, US 2021–2050. *Environmental Research: Infrastructure and Sustainability*, *3*(1), 015013. https://doi.org/10.1088/2634-4505/acc548

OpenStreetMap. (2024). *OpenStreetMap Wiki*. https://wiki.openstreetmap.org/

OurAirports. (2024). *Open data downloads*. https://ourairports.com/data/

Özgenel, M. (2016). *Rural arterial road planning and design steps (Session 12: Roads: Planning, Design and Construction Issues)*. 663–669. http://ijtte.com/uploads/news_files/ICTTE%20Belgrade%202016_Proceedings.pdf

Ramm, F. (2022). *OpenStreetMap Data in Layered GIS Format*. 30.10.2024. https://download.geofabrik.de/osm-data-in-gis-formats-free.pdf

Rebello, T. A. (2022). *Estimating the carbon contribution of the construction and operation of parking spaces in the City of Vancouver*. City of Vancouver. https://sustain.ubc.ca/sites/default/files/2022-057_Estimating%20the%20carbon%20contribution%20of%20parking%20spaces_Ayres%20Rebello.pdf

Schmied, M., & Mottschall, M. (2013). *Treibhausgasemissionen durch die Schieneninfrastruktur und Schienenfahrzeuge in Deutschland* (FKZ 363 01 244). Öko-Institut e.V. https://www.oeko.de/oekodoc/1852/2013-520-de.pdf

Streeck, J., Baumgart, A., Haberl, H., Krausmann, F., Cai, B., Fishman, T., Lanau, M., Berrill, P., Cao, Z., Deetman, S., Frantz, D., Krey, V., Mastrucci, A., Miatto, A., Pauliuk, S., Rousseau, L., Saxe, S., Densley Tingley, D., Ünlü, G., & Wiedenhofer, D. (2024). *Quantifying material stocks in long-lived products: Research frontiers for sustainable resource-use strategies* [Preprint]. SSRN. https://doi.org/10.2139/ssrn.4960374

United Nations Development Programme. (2025). *Human Development Index (HDI)* [dataset]. https://hdr.undp.org/data-center/human-development-index#/indicies/HDI

United Nations Statistics Division. (2024). *Bitumen* [dataset]. https://data.un.org/Data.aspx?d=EDATA&f=cmID%3ABT

Ünlü, G., Maczek, F., Min, J., Frank, S., Glatter, F., Natsuo Kishimoto, P., Streeck, J., Eisenmenger, N., Krey, V., & Wiedenhofer, D. (2024). *MESSAGEix-Materials v1.0.0: Representation of Material Flows and Stocks in an Integrated Assessment Model* [Preprint]. Integrated assessment modeling. https://doi.org/10.5194/egusphere-2023-3035

van der Voet, E., Kleijn, R., Huele, R., Ishikawa, M., & Verkuijlen, E. (2002). Predicting future emissions based on characteristics of stocks. *Ecological Economics*, *41*(2), 223–234. https://doi.org/10.1016/S0921-8009(02)00028-9

Virág, D., Wiedenhofer, D., Baumgart, A., Matej, S., Krausmann, F., Min, J., Rao, N. D., & Haberl, H. (2022). How much infrastructure is required to support decent mobility for all? An exploratory assessment. *Ecological Economics*, *200*, 107511. https://doi.org/10.1016/j.ecolecon.2022.107511

Wiedenhofer, D., Baumgart, A., Matej, S., Virág, D., Kalt, G., Lanau, M., Tingley, D. D., Liu, Z., Guo, J., Tanikawa, H., & Haberl, H. (2024). Mapping and modelling global mobility infrastructure stocks, material flows and their embodied greenhouse gas emissions. *Journal of Cleaner Production*, *434*, 139742. https://doi.org/10.1016/j.jclepro.2023.139742

Wiedenhofer, D., Schug, F., Gauch, H., Lanau, M., Drewniok, M. P., Baumgart, A., Virág, D., Watt, H., Serrenho, A. C., Densley Tingley, D., Haberl, H., & Frantz, D. (2024). Mapping material stocks of buildings and mobility infrastructure in the United Kingdom and the Republic of Ireland. *SSRN Electronic Journal*. https://doi.org/10.2139/ssrn.4670794

Wiedenhofer, D., Steinberger, J. K., Eisenmenger, N., & Haas, W. (2015). Maintenance and Expansion: Modeling Material Stocks and Flows for Residential Buildings and Transportation Networks in the EU25. *Journal of Industrial Ecology*, *19*(4), 538–551. https://doi.org/10.1111/jiec.12216

World Bank. (2024, August 27). *World Bank Country and Lending Groups*. https://datahelpdesk.worldbank.org/knowledgebase/articles/906519

Zeitz, A., Griffin, C. T., & Dusicka, P. (2019). Comparing the embodied carbon and energy of a mass timber structure system to typical steel and concrete alternatives for parking garages. *Energy and Buildings*, *199*, 126–133. https://doi.org/10.1016/j.enbuild.2019.06.047

Zhang, Z., Sun, X., Ding, N., & Yang, J. (2019). Life cycle environmental assessment of charging infrastructure for electric vehicles in China. *Journal of Cleaner Production*, *227*, 932–941. https://doi.org/10.1016/j.jclepro.2019.04.167
